# Genetically Designed Living Bacteria with Melanogenesis for Tumor‐Specific Pigmentation and Therapeutic Intervention

**DOI:** 10.1002/advs.202402709

**Published:** 2024-06-18

**Authors:** Liying Wang, Qi Wu, Qi Lyu, Dan Lu, Lehang Guo, Chao Zhong, Min Wang, Chang Liu, Bolin An, Huixiong Xu, Minfeng Huo

**Affiliations:** ^1^ Department of Medical Ultrasound Shanghai Tenth People's Hospital Shanghai Frontiers Science Center of Nanocatalytic Medicine School of Medicine Tongji University 301 Middle Yanchang Rd. Shanghai 200072 P. R. China; ^2^ Department of Ultrasound Zhongshan Hospital Institute of Ultrasound in Medicine and Engineering Fudan University Shanghai 200032 P. R. China; ^3^ Center for Materials Synthetic Biology Shenzhen Institute of Synthetic Biology Shenzhen Institute of Advanced Technology Chinese Academy of Sciences Shenzhen 518055 P. R. China; ^4^ Shanghai Institute of Ceramics Chinese Academy of Sciences Shanghai 200050 P. R. China

**Keywords:** colorectal cancer, genetically engineered bacteria, immunotherapy, intratumoral pigmentation, photonic hyperthermia

## Abstract

Visual observation and therapeutic intervention against tumors hold significant appeal for tumor treatment, particularly in meeting the demands of intraoperative navigation. From a clinical perspective, the naked‐eye visualization of tumors provides a direct and convenient approach to identifying tumors and navigating during surgery. Nevertheless, there is an ongoing need to develop effective solutions in this frontier. Genetically engineered microorganisms are promising as living therapeutics for combatting malignant tumors, leveraging precise tumor targeting and versatile programmed functionalities. Here, genetically modified *Escherichia coli* (*E. coli*) MG1655 bacterial cells are introduced, called MelaBac cells, designed to express tyrosinase continuously. This bioengineered melanogenesis produces melanin capable of pigmenting both subcutaneous CT26 xenografts and chemically induced colorectal cancer (CRC). Additionally, MelaBac cells demonstrate the initiation of photonic hyperthermia therapy and immunotherapy against tumors, offering promising selective therapeutic interventions with high biocompatibility.

## Graphical abstract

Tyrosinase‐engineered microbes with intrinsic melanogenesis (MelaBac cells) are designed and constructed for tumor‐specific targeting and pigmentation, offering naked‐eye visualization and enabling NIR‐initiated photonic hyperthermia therapeutics against tumors. In addition, these microbes can effectively activate anti‐tumor immunity for prominent immunotherapy with high biocompatibility, presenting multiple therapeutic intervention strategies with high clinical translational promises.

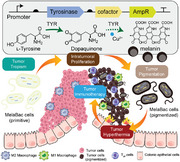

## 1 Introduction

Probiotics have emerged as valuable therapeutic agents, demonstrating significant efficacy in treating various diseases such as cancer,^[^ ^1^ ^]^ metabolic abnormalities,^[^ ^2^ ^]^ and gastrointestinal disease.^[^ ^3^ ^]^ Leveraging developed synthetic biology techniques, these engineered living systems have been further modified to encode functional proteins and peptides,^[^ ^4^ ^]^ transform essential substances,^[^ ^5^ ^]^ and generate valuable drug molecules^[^ ^6^ ^]^ in response to stimuli or continuous workflows. This versatility makes them particularly appealing in the treatment of human chronic illnesses, including innovative approaches to chemoprevention against pathological conditions such as malignant tumors.^[^ ^7^ ^]^ For instance, Nie and colleagues introduced the anti‐CD47 nanobody cargo through heterologous expression within genetically modified bacterial cells. The magnetically controlled lysis of these cells subsequently activates the innate and adaptive antitumor immune responses in synergy with the generated anti‐CD47 nanobodies in the lysate.^[^ ^8^ ^]^ This collaborative action significantly contributes to the robust therapeutic effects against tumors. Additionally, microbial chassis can be strategically designed to produce toxic substances in controlled manners for precise tumor intervention. For example, an ultrasound‐initiated heat‐induced genetic circuit embedded in microbes to produce interferon‐γ molecules exemplifies a responsive approach for tumor immunotherapy.^[^ ^9^ ^]^ In a parallel effort, Chang and colleagues pioneered diet‐initiated antitumor microbes engineered with myrosinase, transforming cruciferous vegetable diet components into sulforaphane.^[^ ^10^ ^]^ These innovative strategies significantly advance genetically engineered microbes for effective and targeted tumor intervention.

In addition to the strategies for tumor intervention, the detection and visualization of malignant tumors play a crucial role in intraoperative navigation and evaluation.^[^ ^11^ ^]^ From a clinical standpoint, naked‐eye visualization of tumors provides a straightforward and convenient approach for observing tumors and navigating during surgery. However, there is a need for the continued development of effective solutions in this realm. To address these challenges, a method involving the induction of tumor‐targeting intratumoral pigment production has been proposed. Melanin, a natural pigment found in versatile human tissues, has been shown to provide biocompatible contrasted visualization of tumor tissue and enables near‐infrared (NIR)‐initiated photonic hyperthermia therapeutics against tumors.^[^ ^12^ ^]^ Hence, incorporating the photoacoustic imaging modality, accompanied by the optical absorption properties of melanin, presents significant potential for both tumor diagnosis and intervention.

Here, we present an approach emphasizing naked‐eye visualization for direct and convenient tumor observation and intraoperative navigation. Inspired by melanocytes, we utilized melanin for biocompatible contrasted visualization of tumor tissue. Genetically engineered *E. coli* MG1655 bacterial cells, termed MelaBac cells, were designed to constantly express the tyrosinase enzyme, facilitating efficient melanogenesis under copper ions and tyrosine substrates. The facultative anaerobic nature of MelaBac cells enables effective and sustained tumor targeting and retention, resulting in prominent intratumoral pigmentation for naked‐eye visualization. This innovative approach was successfully applied to subcutaneous tumor xenografts and sporadic tumors such as chemical‐induced CRC, facilitating photonic hyperthermia therapeutics and activating robust anti‐tumor immunity against tumors, demonstrating high biocompatibility. In summary, our work presents multiple therapeutic intervention strategies based on genetically engineered bacterial cells, leveraging intratumoral pigmentation for naked‐eye visualization and effective tumor immunotherapy.

## 2 Results and Discussion

### 2.1 Construction and Melanogenesis of MelaBac Cells

**Figure 1:**
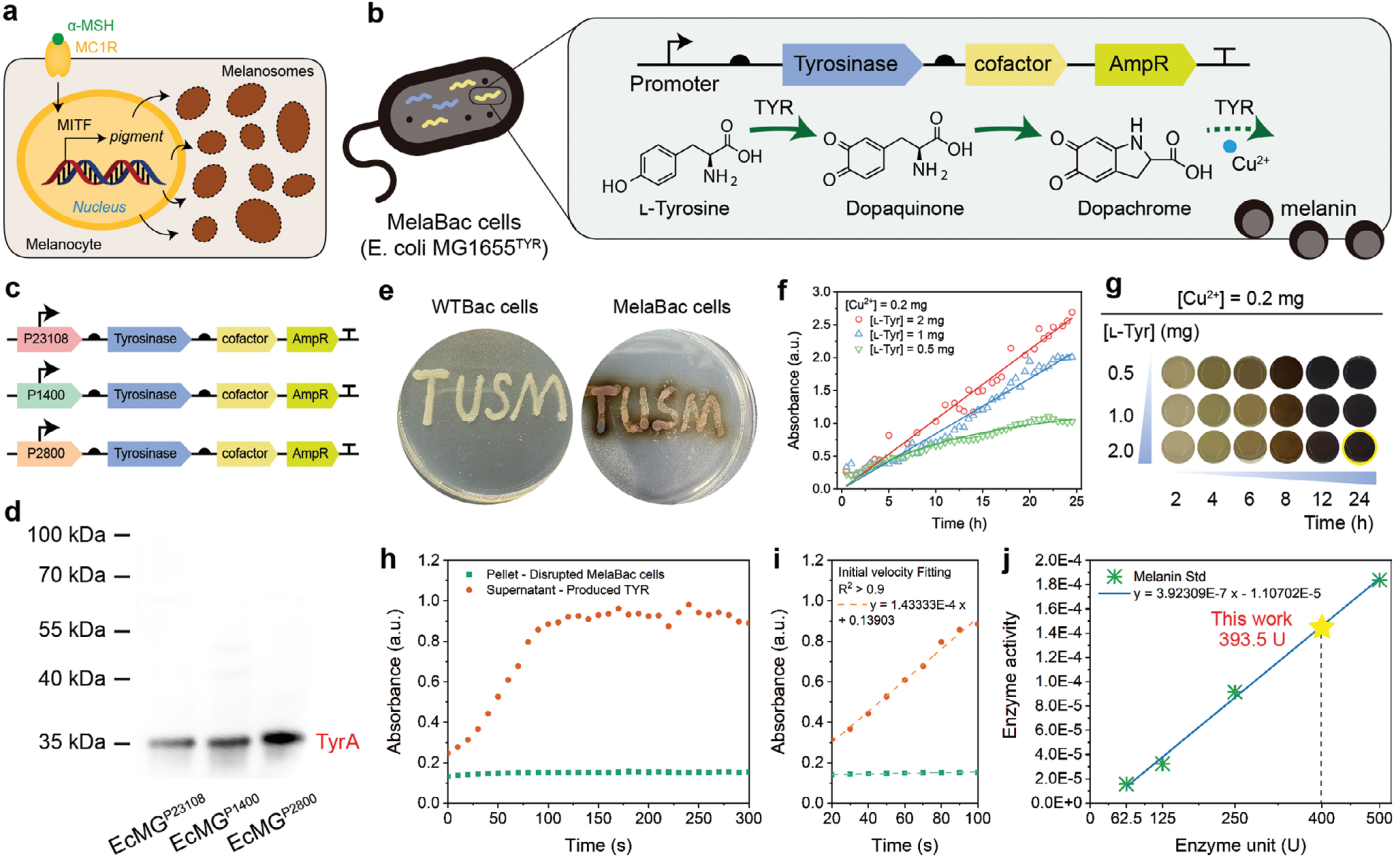
Construction of MelaBac cells and their melanogenesis kinetics. a) Schematic illustration of the melanogenesis process by melanocytes. b) Gene circuit illustration of the genetically engineered MelaBac comprised of the promoter, TYR, cofactor, and the ampicillin resistance gene. The transformed MelaBac cells could perform melanogenesis with copper and l‐tyrosine substrate. c) Gene circuit illustration of MelaBac cells with different promoters (pJ23108, p1400, and p2800). d) Western blot for histag‐labeled TYR identification of EcMG^TYR^‐pJ23108, EcMG^TYR^‐p1400, and EcMG^TYR^‐p2800. e) Plating patterns of WTBac cells and MelaBac cells. f,g) Time‐course optical absorbance f) and corresponding digital photographs g) of LB medium containing EcMG^TYR^‐p2800 (10^8^ c.f.u.) in the presence of different concentrations of l‐tyrosine (0.5, 1, and 2 mg) and copper ions (0.2 mg) along time (2, 4, 6, 8, 12, and 24 h). Data are presented as they are. h) Melanogenesis chromogenic curve for the pellet or supernatant of the disrupted MelaBac cells. Data are presented as they are. i) Initial velocity curve and corresponding fitting of the chromogenic curve. Data are presented as they are. j) Calculation of the enzymatic activity of the MelaBac cell lysate with the calibration of the tyrosinase standard. Data are presented as they are.

Melanocytes can produce the melanin pigment through a process called melanogenesis. Upon the stimulation of bioactive peptides α‐melanocyte stimulating hormone (α‐MSH) to the melanocortin 1 receptor (MC1R) of melanocytes, the biosynthesis of melanin is activated by microphthalmia‐associated transcription factor (MITF). It coordinates the tyrosinase (TYR) and associated proteins and substances for eumelanin synthesis, assembling the melanosomes inside the melanocytes (**Figure** **1**a).^[^ ^13^ ^]^ To imitate such a melanogenesis process in prokaryotic chassis *E. coli*, we have constructed an ampicillin‐resistant plasmid containing constitutive promoters, TYR‐encoding fragments, and associated cofactor fragments through Gibbson assembly (Figure S1, Supporting Information). The genetically engineered strain was designed to catalyze l‐tyrosine to melanin under the presence of Cu^2+^ (Figure 1b). We then characterize the process of melanogenesis in the solution. When recombinant tyrosinase (250 U) was supplemented in the solution containing the l‐tyrosine substrate (20 µg, 1 mL), the colorless solution immediately turned to an orange appearance due to the production of dopachrome (λ_max_ = 475 nm) in 1 min (Figure S2, Supporting Information). We then added Cu^2+^ (0.4 mg) into the above solution and found that the orange appearance gradually decayed and turned to a brown appearance in 10 min, implicating further polymerization process from dopachrome to melanin (Figure 1b; Figure S2, Supporting Information).

To optimize the expressions of TYR and efficiency of the melanogenesis performance, we selected one moderate‐expressing promoter (pJ23108), and two high‐expressing constitutive promoters, p1400^[^ ^14^ ^]^ and p2800,^[^ ^14^ ^]^ to construct the engineered bacterial strains, forming EcMG^TYR^‐pJ23108, EcMG^TYR^‐p1400, and EcMG^TYR^‐p2800 strains, respectively (Figure 1c). It is important to highlight that the designations for p1400 and p2800 were SLP2018‐2‐101 and SLP2018‐2‐167 promoters respectively. These two promoters were derived from the recently developed Nonrepetitive Parts Calculator^[^ ^14^ ^]^ and demonstrated ≈1400 and 2800 times the expression level of sfGFP compared to the widely used pJ23100 Anderson constitutive promoter. Consequently, we named these two promoters p1400 and p2800, reflecting their high expression levels. Through western blot analysis of these strains using an anti‐Histag antibody, protein bands with molecular weights of ≈35 kDa could be observed for all the lanes from three EcMG^TYR^ strains, corresponding to encoded TyrA proteins with a theoretical molecular weight of 30.74 kDa (Figure 1d). Western blot quantification also verified the highest TyrA protein expression of EcMG^TYR^‐p2800 strains (Figure S3, Supporting Information). MelaBac cells can grow and pigmentate in the designed pattern on the LB agar plate containing Cu^2+^ (0.05 mg mL^−1^) and l‐tyrosine (0.5 mg mL^−1^). The inability of WTBac cells to pigmentate the pattern can also be indicated (Figure 1e). We next evaluate the melanogenesis performance via time‐dependent pigmentation by adding Cu^2+^ and l‐tyrosine with varied concentrations. The melanogenesis process generally occurred in 6 h post‐incubation of the engineered strains and substrates. Darker pigmentation could be observed for EcMG^TYR^‐p2800 strains in 8–12 h compared to the other strains (Figure S4, Supporting Information). Featuring the most potent melanogenesis performance, the EcMG^TYR^‐p2800 strain was selected for further research (denoted as the MelaBac cells thereafter). We also investigate the optimized concentration of Cu^2+^ and l‐tyrosine substrate further. We found that MelaBac could effectively generate the melanin pigment along with the increasing Cu^2+^ (from 0.05 to 0.2 mg) and l‐tyrosine (from 0.5 to 2 mg) over time (Figure 1f,g; Figure S5, Supporting Information). With standard calibrations according to UV‐vis spectroscopy, the end‐point generation of melanin was quantified as 62.69 µg mL^−1^ under specific conditions of 0.2 mg Cu^2+^, 2 mg l‐tyrosine, and a pigmentation time of 24 h (Figure S6, Supporting Information). With calibration of the standard tyrosinase chromogenic activities (Figure S7, Supporting Information), we have determined the enzymatic activity of the MelaBac cells to be 393.5 U per 10^7^ c. f. u. cells after homogeneously disrupting the MelaBac cells through ultrasonic destruction under 0 °C (Figure 1h–j).

**Figure 2:**
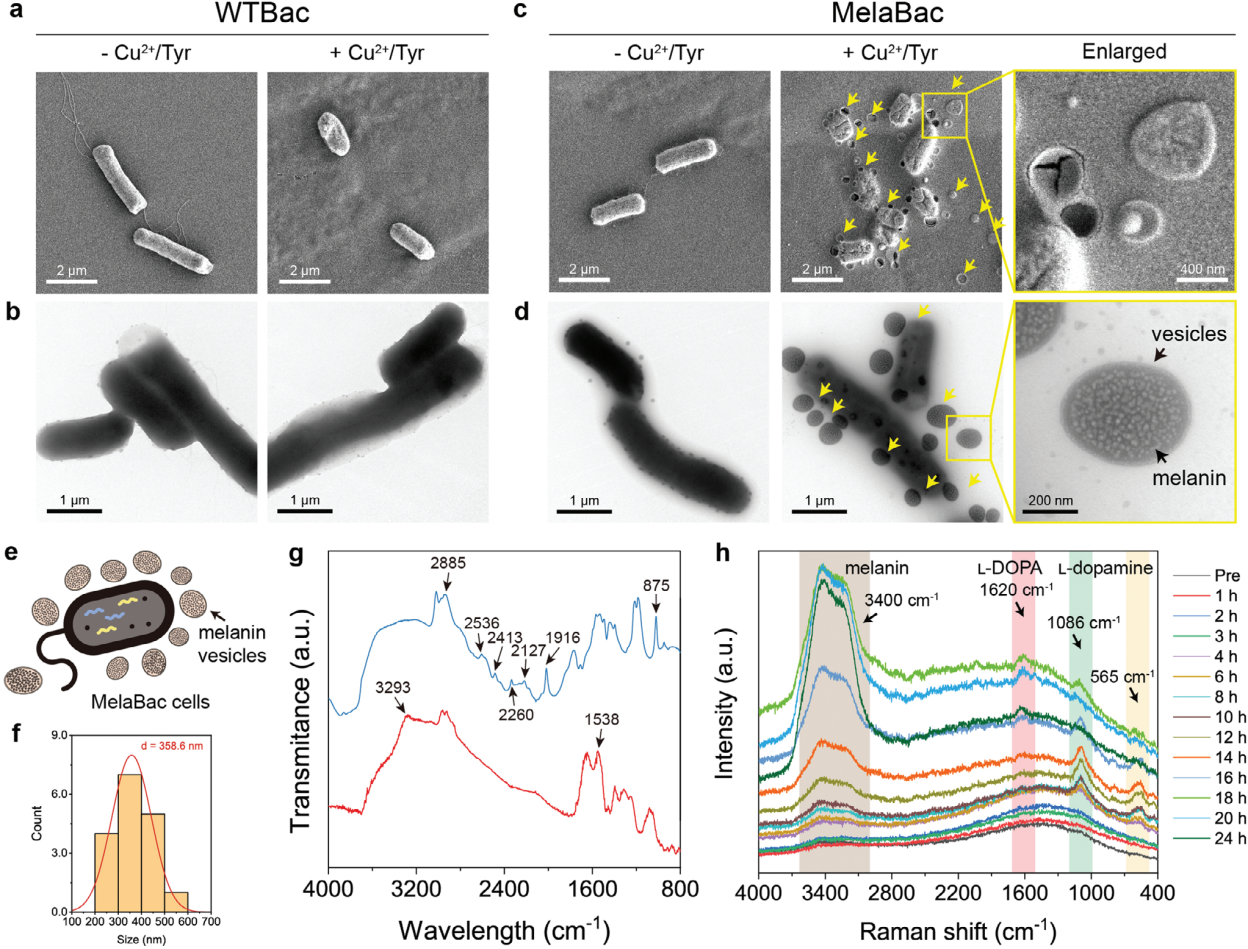
Characterizations of the melanogenesis of MelaBac cells. a,b) SEM a) and TEM b) images of EcMG^WT^ cells with and without Cu^2+^/l‐tyrosine. c,d) SEM c) and TEM d) images of EcMG^TYR^‐p2800 cells with and without Cu^2+^/l‐tyrosine. e) Schematic of the secretion of melanin vesicles by MelaBac. f) Statistical distribution of the diameter profile of the generated melanin vesicles. Data are presented as they are. g) FTIR spectra of MelaBac with (blue) and without (red) melanin generation. h) In situ Raman spectra of MelaBac cells during the melanogenesis at varied time points.

We also inspect the morphology of the wild‐type bacterial cells (WTBac) and MelaBac cells with or without the addition of copper/l‐tyrosine. It could be observed from Scanning Electron Microscopic (SEM) and Transmission Electron Microscopic (TEM) images that WTBac and MelaBac exhibit typical rod‐shaped bacterial cells (**Figure** **2**a–d). In the presence of copper/l‐tyrosine, spheroid vesicles could be observed surrounding the MelaBac cells. These vesicles, with an average diameter of 358.6 nm, are suggested to be the secretion pathway of intracellular melanin pigments (Figure 2e,f). The pigmentated MelaBac cells were dehydrated by graded ethanol for further TEM observation. It has been verified that larger vesicles could be observed in bacterial pellets while smaller vesicles were suspended in the supernatant (Figure S8, Supporting Information). We also characterized the primitive and pigmentated MelaBac cells using Fourier transform infrared (FTIR) spectroscopy. Additional peaks emerged on the spectra of MelaBac cells with pigmentation compared to the primitive MelaBac cells. These peaks were explicitly assigned to the vibrations of melanin pigments (Figure 2g). We also inspect the pigmentation process of MelaBac cells using in situ Raman spectroscopy at varied time points to monitor the production of the substances. In 4 h, no Raman‐active substances could be observed. Raman peaks at 565, 1086, and 3400 cm^−1^ emerged in 6 h post‐coincubation of the MelaBac cells with Cu^2+^ and l‐tyrosine. Peaks at 565 and 1086 cm^−1^ originate from the vibration of l‐dopamine.^[^ ^15^ ^]^ Meanwhile, peaks at 3400 cm^−1^ are assigned explicitly as the vibration of the hydroxyl groups of melanin pigment. As the time prolongs to 16 h, the amplitude of the peaks (565 and 1086 cm^−1^) decreased while a new Raman peak positioned at 1620 cm^−1^ emerged, implicating the transformation from l‐dopamine to l‐DOPA. For the peak positioned at 3400 cm^−1^, constant and sustained melanin production within 24 h could be indicated (Figure 2h). These results are consistent with the reaction pathway we imitate at the solution level.

### 2.2 Pigmentated MelaBac Cells Initiated the Hyperthermia Immunotherapeutics Against Colon CT26 Cells In Vitro

**Figure 3:**
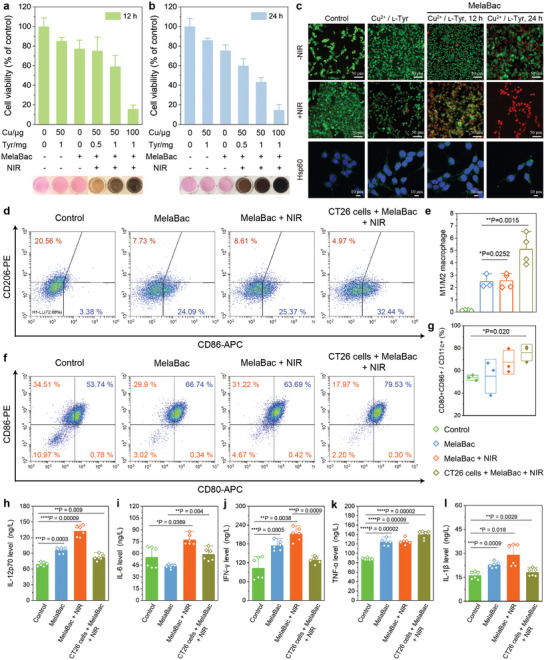
In vitro cellular experiment and antitumor immunity. a,b) Cell viability of CT26 with different treatments in 12 h a) or 24 h b) as indicated. Data are presented as mean ± SD, *n* = 4. c) Confocal microscopic images of Calcein‐AM/PI stained CT26 cells with different treatments as indicated. d,e) Flow cytometric analysis of CD86^+^/CD206^+^ d) and corresponding statistical analyses of the M1/M2 ratio e) of RAW264.7 macrophages with treatments of MelaBac, MelaBac + NIR and CT26 cells + MelaBac + NIR. Data are presented as they are and mean ± SD, *n* = 4. Significances were analyzed by student's *t*‐test. ^*^ *p* < 0.05, ^**^ *p* < 0.01. f,g) Flow cytometric analysis of CD80^+^CD86^+^ f) and corresponding statistical analyses of the CD80^+^CD86^+^/CD11c^+^ ratio g) of DCs with treatments of MelaBac, MelaBac + NIR and CT26 cells + MelaBac + NIR. Data are presented as they are and mean ± SD, *n* = 3. Significances were analyzed by student's t‐test. ^*^ *p* < 0.05. h–l) ELISA analyses of IL‐12p70 h), IL‐6 i), IFN‐γ j), TNF‐α k), and IL‐1β l) cytokines secreted by DCs. Data are presented as they are and mean ± SD, *n* = 6. Significances were analyzed by student's *t*‐test. ^*^ *p* < 0.05, ^**^ *p* < 0.01, ^***^ *p* < 0.001, and ^****^ *p* < 0.0001.

When CT26 cells were co‐incubated with MelaBac cells in the absence of Cu^2+^/l‐tyrosine substrate, a minor reduction of the cell viability could be observed, possibly due to the nutrition competition (**Figure** **3**a,b). In the presence of Cu^2+^/l‐tyrosine substrate, the melanin production of MelaBac cells pigmentates the medium substances with optical absorptions in the first NIR region (750–850 nm) in 12 and 24 h, further enabling the laser‐exited hyperthermia therapeutic against the murine colon CT26 tumor cells. The photonic hyperthermia performance of pigmentated MelaBac cells in the 1640 medium was initially evaluated. Medium solutions containing MelaBac (10^7^ c.f.u.), Cu^2+^, and l‐tyrosine at varied concentrations were allowed to pigmentate for 12 and 24 h, respectively. In 12 h, these pigmentated MelaBac cells can elevate the temperature of the solution by 7.9, 12.9, and 19.8 K, respectively, under the 808 nm laser irradiation (1 W cm^−2^, 8 min). When the pigmentation time was prolonged to 24 h, the temperature of these solutions increased by 18.4, 18.7, and 20.9 K, respectively, under identical laser parameters (Figure S9, Supporting Information). NIR irradiation (808 nm, *P* = 1 W cm^−2^, *t* = 8 min) of the culture at 24 h led to a substantial decline in the viability of CT26 cells (40.0% for Cu^2+^/l‐tyrosine of 50 µg/0.5 mg; 85.1% for Cu^2+^/l‐tyrosine of 100 µg/1 mg) (Figure 3a,b). The temperature elevation curve and the viability of the cells with NIR irradiation were also evaluated. Minor temperature elevation and non‐significant cytotoxicity could be found (Figure S10, Supporting Information). The live/dead cell distribution was further visualized by Calcein‐AM/PI dual staining and confocal microscopic observations. Destruction of the CT26 tumor cells could be identified in the group treated with pigmentated MelaBac and NIR irradiation (Figure 3c).

Macrophages with different phenotypes play distinct characters in innate antitumor immunity. The M1‐phenotype of macrophages has shown potent intrinsic bactericidal and antitumor immunity performance, while the M2‐phenotype of macrophages is particularly immunosuppressive.^[^ ^16^ ^]^ To identify the macrophage polarization upon different stimulations, a population of CD86 (M1 feature marker) or CD206 (M2 feature marker) positive murine RAW264.7 macrophages have been quantified using flow cytometry. Untreated RAW264.7 macrophages have 3.38% of the M1‐phenotype population and 20.56% of the M2‐phenotype population, respectively. The M1‐phenotype population increased to 24.09%, 25.37%, and 32.44% for macrophages treated in MelaBac, MelaBac + NIR, and CT26 cells + MelaBac + NIR groups, respectively, along with the decreasing population percentages of M2‐phenotype. These statistics suggest that the destruction of tumor cells by hyperthermia could intensively trigger macrophage polarization toward the M1 phenotype (Figure 3d,e). We also evaluate whether MelaBac pigmentation‐enabled NIR hyperthermia could induce adaptive immunity by quantifying the percentage of dendritic cell (DC) maturation (CD80^+^CD86^+^). In a Transwell setup, DCs extracted from bone marrows were inoculated in the lower chamber of the Transwell and co‐incubated with different treatments (MelaBac, MelaBac + NIR, CT26 + MelaBac + NIR) in the upper chamber, respectively (Figure S11, Supporting Information). According to the CD80^+^CD86^+^ percentages, the DCs maturation was determined as 66.74%, 63.69%, and 79.53%, compared to 53.74% in the untreated group (Figure 3f,g). The increased secretion of IL12p70, IL‐6, IFN‐γ, TNF‐α, and IL‐1β cytokines additionally validates the activation and maturation process of DCs (Figure 3h–l).^[^ ^17^ ^]^ DCs maturation endows the antigen processing and presentation to stimulate the activation of adaptive antitumor immunity,^[^ ^18^ ^]^ presenting the therapeutic promises of the immunotherapy.

### 2.3 In Vivo Subcutaneous Tumor Therapeutics and Hemodynamic Modulation Enabled by MelaBac Cells

Before investigating the in vivo tumor therapeutic performance enabled by MelaBac cells, tumor targeting performance was initiated by conducting tissue biodistribution assays. All in vivo animal experiments have been approved by the Laboratory Animal Center of Shanghai Tenth Peoples’ Hospital (license: SHDSYY‐2023‐6600). Murine colon CT26 cells were initially inoculated in the mice for subcutaneous tumor xenograft establishment. All mice were administrated with MelaBac cells (1 × 10^8^ c.f.u. in 100 µL saline, i.v.) and euthanized at predetermined time points (2, 6, 12, 24, and 48 h). Tumor xenografts and major organs were dissected, homogenized, and plated into the selected agar plates to inoculate the remaining bacterial cells. The plating results showed that the MelaBac cells were majorly accumulated in the liver in 2 h and gradually eliminated in 12 h. Accumulation inside the tumor can be observed at 6 h post‐administration. These tumor‐accumulated bacterial cells can grow and stay even in 48 h post‐administration, validating the sustained and robust tumor‐targeting performance of MelaBac cells toward tumor tissue (Figures S12–S13, Supporting Information). Colony counting results further revealed that MelaBac titer within tumor tissues reaches the maximum of 5.55 × 10^5^ c.f.u. in 24 h post injection (Figure S14, Supporting Information) while liver tissues were found with a maximum of 740 c.f.u. in 2 h post injection. A tumor/liver accumulation ratio of 750 could be calculated, implicating the tumor‐selective targeting performance of MelaBac cells.

**Figure 4:**
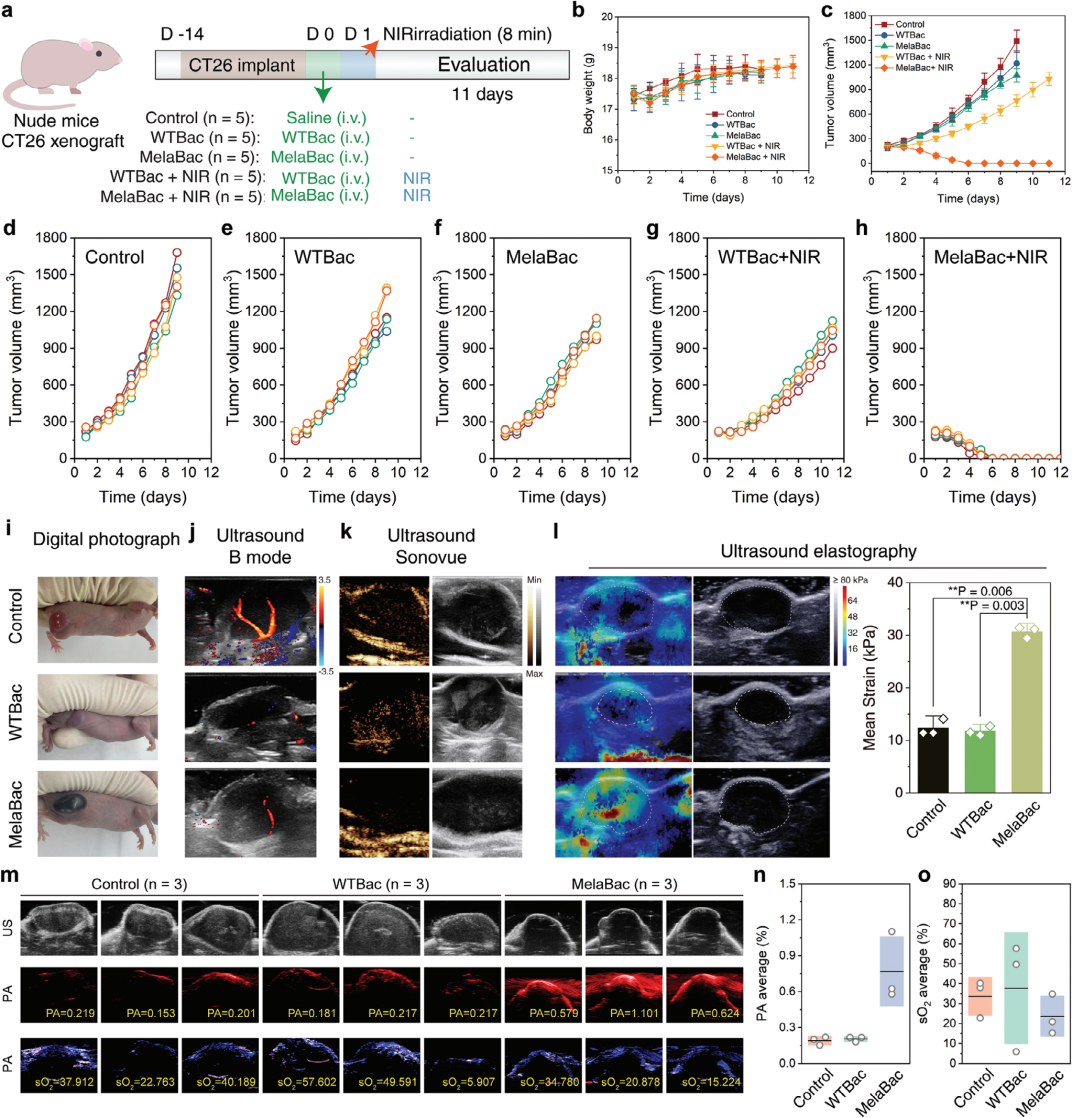
In vivo, therapeutics and hemodynamic modulation enabled by MelaBac cells. a) Treatment schedule of CT26‐xenograft bearing nude mice after WTBac or MelaBac injection with or without NIR irradiation. b,c) b) Body weight, and c) tumor volume curves of mice from control and different treatment groups. Data are presented as mean ± SD, *n* = 5. d–h) Tumor volume curves of each mouse from different treatment groups. Data are presented as they are. i–l) i) Digital photographs, j) ultrasound B mode images, k) ultrasound Sonovue mode images, and l) Ultrasound elastography imaging and corresponding statistical analysis of the mean strain of the xenografts of mice from control, WTBac, and MelaBac groups. Data are presented as they are and mean ± SD, *n* = 3. Significances were analyzed by student's *t*‐test. ^**^ *p* < 0.01. m) Ultrasound, PA, and O_2_ saturation images of CT26 tumor‐bearing nude mice 24 h post saline injection, WTBac, or MelaBac (*n* = 3). n,o) Corresponding statistical analysis of the n) PA values and o) sO_2_ values. Data are presented as they are and mean ± SD.

The anti‐tumor performance of in situ pigmentation of MelaBac cells was further validated in CT26‐xenograft tumor‐bearing nude mice. These mice were randomly divided into five groups including control (saline, 100 µL), WTBac (1 × 10^8^ c.f.u. in 100 µL saline, i.v.), MelaBac (1 × 10^8^ c.f.u. in 100 µL saline, i.v.), WTBac + NIR (1 × 10^8^ c.f.u. in 100 µL saline, i.v., 808 nm, *P* = 1 W cm^−2^, *t* = 8 min) and MelaBac + NIR (1 × 10^8^ c.f.u. in 100 µL saline, i.v., 808 nm, *P* = 1 W cm^−2^, *t* = 8 min) (**Figure** **4**a). During the evaluation period, the body weight of mice exhibited a slight decrease after WTBac or MelaBac administration. It recovered rapidly in 2 days (Figure 4b), indicating the biosafety of WTBac and MelaBac cells after intravenous injection at a dose of 10^8^ c.f.u. bacterial cells. Upon MelaBac cell administration, the xenografts of mice were pigmentated effectively in 2 days, compared to the control group and WTBac injection groups. The xenograft pigmentation validates the tumor‐targeting performance and intratumoral pigmentation process enabled by MelaBac cells. For hyperthermia evaluation under NIR irradiation, we found that the temperature of tumor xenografts of untreated mice exhibited a slight temperature elevation of 7.3 °C (Figure S15, Supporting Information). The tumor temperature of mice in the WTBac + NIR group could reach 46.9 °C after 808 nm laser irradiation (1 W cm^−2^, 8 min). Such a mild hyperthermia effect was insufficient to restrain the tumor growth completely. For the MelaBac + NIR group, the xenograft temperature could reach 59.7 °C, destroying the tumor tissue with high efficacy (Figure S16, Supporting Information). For the therapeutic outcome, treatment of WTBac or MelaBac cells slightly impeded the rapid growth of the tumor. While treatment of WTBac cells and NIR irradiation further inhibited tumor proliferation. With the combination of MelaBac and NIR irradiation, whole tumor destruction could be achieved in 6 days, suppressing the regrowth of the xenograft afterward (Figure 4c–h). In addition, bacteria titer within tumor tissues in 24 h post administration before and after NIR treatment was also identified. MelaBac counting decreased from 8.31 × 10^5^ c.f.u. g^−1^ to 3.6 × 10^4^ c.f.u. g^−1^ while WTBac decreased from 8.41 × 10^5^ c.f.u. g^−1^ to 6.47 × 10^5^ c.f.u. g^−1^ (Figure S17, Supporting Information), indicating that the pigmentation of MelaBac‐mediated hyperthermia therapy could also kill intratumoral MelaBac and facilitate further immune responses.

Intratumoral accumulation of the MelaBac cells may potentially regulate the hemodynamic status of the tissue. Therefore, ultrasound imaging technologies, including blood‐flow‐based color Doppler flow imaging (CDFI), Sonovue contrast imaging, stiffness‐based elastography, and photoacoustic imaging, were conducted to evaluate tumor vasculature, stiffness changes, and tumor‐specific melanin accumulation conditions. For another batch experiment of CT26‐xenograft tumor‐bearing nude mice, the digital photograph of the xenograft from the control group reveals a rich blood supply in the xenograft. With WTBac cell administration, the xenograft of mice from the WTBac group turned pale grey due to the intratumoral bacterial accumulation and subsequent thrombosis condition. For the MelaBac cell administration group, the xenograft of mice was deeply pigmentated with a black appearance due to the MelaBac accumulation inside the tumor and the intratumoral melanin production (Figure 4i). From the CDFI images, the CT26 tumor xenograft of mice from the control group was supplied by two major vessels. WTBac or MelaBac treatment typically induces blockade of blood supply (Figure 4j). The contrast images also validate sufficient blood supply in the xenograft of the control group while decreased or blocked blood supply in the xenograft of the WTBac and MelaBac group, as visualized with Sonovue. The grayscale ultrasonography also indicates potential blood clots in the control group and calcification sites in the WTBac and MelaBac groups (Figure 4k). The increased tumor stiffness after MelaBac treatment in elastography observation reveals the sign of inflammation possibly attributed to intratumoral bacterial metabolism and immune response (Figure 4l). Photoacoustic imaging and statistical analysis exhibit typically enhanced signal intensity in the MelaBac group, verifying in situ melanin production compared to the control and WTBac groups (Figure 4m–o).

### 2.4 In Vivo Subcutaneous Tumor Therapeutics and Hematic Investigations

**Figure 5:**
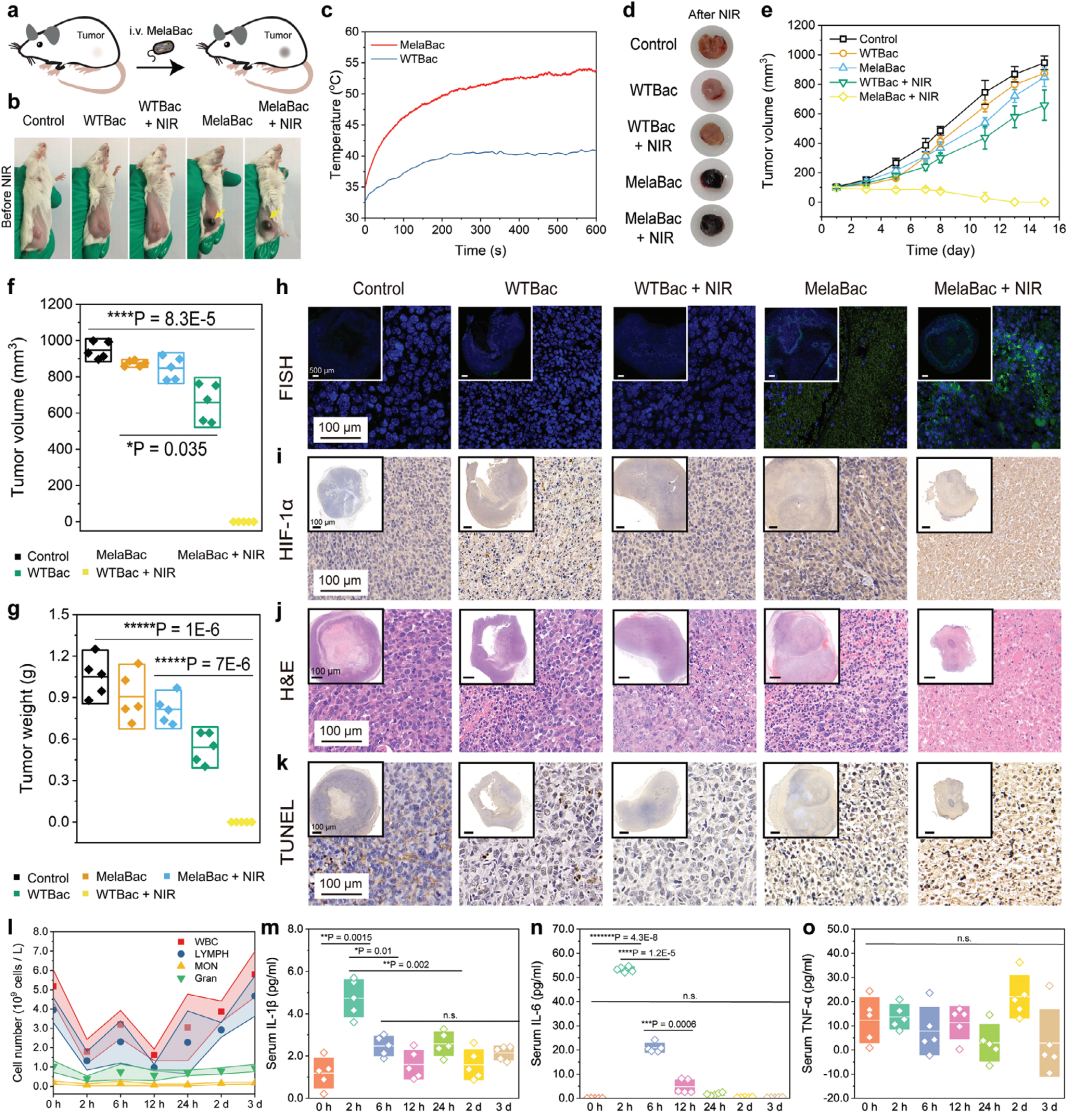
In vivo subcutaneous tumor therapeutics and hematic investigations. a) Schematic illustration of in vivo tumor therapeutics enabled by MelaBac cells and NIR eradication. b) Digital photographs of mice recorded on Day 3. c) Temperature curve of mice from WTBac + NIR and MelaBac + NIR group with NIR irradiation for 10 min. d) Digital photographs of the dissected xenografts of mice from different groups: Control, WTBac, WTBac + NIR, MelaBac, and MelaBac + NIR. e) The xenograft volume curve of mice from different groups during the evaluation timeframe of 15 days. Data are presented as mean ± SD, *n* = 5. f,g) Statistical comparison and analyses of f) tumor volume and g) tumor weight of the dissected xenografts of mice from different groups at the end of the evaluation timeframe. Data are presented as they are and mean ± SD, *n* = 5. Significances were analyzed by student's *t*‐test. ^*^ *p* < 0.05, ^****^ *p* < 0.0001, ^*****^ *p* < 0.00001. h) Confocal microscopic images of FISH probe (specific for MelaBac cells) stained tumor sections of mice from different groups. i) Histological microscopic images of HIF‐1α‐stained tumor sections of mice from different groups. j,k) Histological images of j) H&E‐stained and k) immunohistochemical images of TUNEL‐stained tumor sections of mice from different groups. l) Time course blood routine analyses (WBC, LYMPH, MON, and Gran) of mice with MelaBac injections. Timepoints: 0 h, 2 h, 6 h, 12 h, 24 h, 2 d, and 3 d. Data are presented as mean ± SD m–o) Time‐course ELISA assay of m) IL‐1β, n) IL‐6, and o) TNF‐α of the serum of mice with MelaBac injection. Timepoints: 0 h, 2 h, 6 h, 12 h, 24 h, 2 d, and 3 d. Data are presented as they are and mean ± SD, *n* = 5. Significances were analyzed by student's *t*‐test. ^*^ *p* < 0.05, ^**^ *p* < 0.01, ^***^ *p* < 0.001, ^****^ *p* < 0.0001, ^*******^ *p* < 0.0000001, n.s. for non‐significant.

To investigate the in vivo antitumor potentials and immunity‐associated therapeutic consequence enabled by MelaBac cells, twenty‐five Balb/c mice bearing CT26 subcutaneous xenografts have been allocated into five groups randomly: Control (saline), WTBac group (WTBac, 1 × 10^8^ c.f.u. in 100 µL saline, i.v.), WTBac + NIR group (WTBac, 1 × 10^8^ c.f.u. in 100 µL saline, i.v., 808 nm laser irradiation in 24 h post‐injection, *P* = 1 W cm^−2^, *t* = 10 min), MelaBac group (WTBac, 1 × 10^8^ c.f.u. in 100 µL saline, i.v.) and MelaBac + NIR group (MelaBac, 1 × 10^8^ c.f.u. in 100 µL saline, i.v., 808 nm irradiation in 24 h post‐injection, *P* = 1 W cm^−2^, *t* = 10 min) (**Figure** **5**a). Mice were fed with an irradiated Diet provided by Jiangsu Xietong Pharmaceutical Bio‐engineering Co., Ltd (Product code:1 010 065), which contains Cu^2+^ of 17 mg kg^−1^ and l‐tyrosine of 6 g kg^−1^. Copper and tyrosine content within serum and tumor tissues were analyzed. Mice in the control group were detected with a mean tyrosine concentration of 6.04 µm in serum and 79.5 nmol in 1 g of tumor tissue (Figure S18a,b, Supporting Information). Tyrosine concentration decreased to 4.04 µm in serum and 26.94 nmol g^−1^ within tumors due to MelaBac‐mediated pigmentation using tyrosine. We also found that copper concentration in serum and tumor tissue of mice remained non‐significantly changed after MelaBac cells administration as compared to the control group. Intratumoral copper concentration was assayed to be ≈9.98 µg g^−1^ tumor for mice in MelaBac group (Figure S18c,d, Supporting Information). These statistics provide fundamentals for tumor pigmentation enabled by MelaBac. On day 3, in situ tumor pigmentation could be observed for the MelaBac treatment group rather than the WTBac treatment group, implicating that MelaBac cells could effectively accumulate inside the tumor xenograft, providing tanning annotation of the tumor with intense optical absorption for NIR irradiation (Figure 5b). Localized laser treatment of the tumor xenograft induced temperatures of 40.8 and 53.8 °C, respectively, for mice in the WTBac + NIR group and MelaBac + NIR group (Figure 5c). We also observe tumor necrosis of the xenograft of mice in the MelaBac + NIR group, which is different from the tanning annotation we observed in the mice of the MelaBac group (Figure 5d). The xenograft dimensions of mice from different groups were measured every other day. We found that the xenografts of mice from WTBac, WTBac + NIR, and MelaBac groups grew rapidly to reach the ultimate tumor volume of 874.2, 658.5, and 847.8 mm^3^, respectively, in two weeks. For xenograft of mice from the MelaBac + NIR group, complete tumor eradication could be observed (Figure 5e).

After the evaluation timeframe, xenografts were dissected, measured, and weighted. Both tumor volume and weight of mice from the MelaBac + NIR group reduced significantly compared to control and other treatment groups (Figure 5f,g). The dissected xenografts were further sliced into ultrathin sections for immunological and histological inspections. To indicate the intratumoral accumulation of MelaBac cells, 16S ribosomal DNA sequence (5′‐FITC‐TCCACGGCTTCATAGTTTCATTTAAGTCCACC‐3′) was designed to selectively label MelaBac cells via fluorescence in situ hybridization (FISH) analysis of the tumor sections. MelaBac cells were found only in tumors of mice from MelaBac and MelaBac + NIR group, distributed around the nuclei of the tumor cells (Figure 5h). From the immunohistochemical staining of hypoxia‐inducible factor‐1α (HIF‐1α), positive nuclei could be observed for the tumor section from WTBac, MelaBac, and MelaBac + NIR treatment groups, suggesting the intravenous injection of bacterial cells may lead to the formation of thrombus, restricting tumor nutrient and oxygen supply (Figure 5i). In addition, hematoxylin and eosin (H&E) stained tumor sections indicate the most effective necrosis and significantly reduced cell nuclei from the MelaBac + NIR group compared to control and other treatment groups (Figure 5j). Potent cell apoptosis was induced through MelaBac + NIR treatment, as revealed by the terminal deoxynucleotidyl transferase dUTP nick end labeling (TUNEL), validating the effectual therapeutic consequences in combining the in situ MelaBac‐enabled tumor melanogenesis and subsequent laser eradication (Figure 5k). For innate immunity activation, macrophages of M1 phenotype (CD86+CD206‐) increased statistically from 3.70% to 19.40% after MelaBac pigmentation‐mediated photothermal therapy, as compared to the control group (Figure S19, Supporting Information). We also observed that the percentage of matured DCs (CD80+CD86+) in lymph nodes increased from 3.96% to 27.6% in MelaBac group, suggesting the activation of adaptive antitumor immunity (Figure S20, Supporting Information). In addition, T cells within spleen and tumor tissue were isolated and analyzed. For lymphocytes in spleen, the percentage of CD8+ T cells in CD3+ T cells increased from 6.85% in control group to 17.96% in MelaBac + NIR group. (Figure S21, Supporting Information). Analysis of CTLs in tumors reveals that the percentage of CD8+ T cells in CD3+ T cells also increased from 4.47% in control group to 33.6% in MelaBac + NIR group (Figure S22, Supporting Information), collectively validating that MelaBac + NIR treatment induced effective cytotoxic T cell anti‐tumor immunity and macrophages‐mediated innate immunity. Furthermore, T cells isolated from lymph nodes were also collected and analyzed, which were found with a CD8+ T cell percentage elevation from 6.0% in control group to 24.6% in MelaBac + NIR group (Figure S23, Supporting Information). Collectively, MelaBac mediated tumor pigmentation and hyperthermia successfully activated both innate and adaptive immunity effectively.

The plasma samples were also collected from mice for blood biochemical and serum cytokines analysis to investigate the hematic diagram. The inflammatory‐related indexes, including white blood cells (RBC), lymphocytes (LYMPH), monocytes (MON), and granulocytes (Gran), experienced a transient decrease after intravenous injection of MelaBac cells (1 × 10^9^ c.f.u.) within 24 h, and recovered gradually to the physiological level in 3 d (Figure 5l). Other plasma indices, including platelet (PLT), hemoglobin (HGB), hematocrit (HCT), mean corpuscular hemoglobin (MCH), mean corpuscular volume (MCV), red cell distribution (RDW), procalcitonin (PCT), and mean platelet volume (MPV) underwent slight fluctuation and remained basically balanced (Figure S24, Supporting Information). Serum biochemical indices revealing liver and kidney functions also exhibit non‐significant fluctuations, including alkaline phosphate (ALP), alanine transaminase (ALT), aspartate transaminase (AST), urea and creatine (CRE) (Figure S25, Supporting Information). Furthermore, systemic peripheral anti‐tumor immunity condition was evaluated. Both serum interleukin‐1β (IL‐1β) and IL‐6 increased in 2 h post‐injection of MelaBac cells and gradually decreased in 12 h. These pro‐inflammatory cytokines were recovered to physiological levels in 3 d. From these plasma indices, we validate that the MelaBac challenge against mice induces acute inflammatory status in 2 h. The cytokine storm can then be calmed down normally in 12 h, maintaining the healthy conditions afterward (Figure 5m,n). Serum tumor necrosis factor (TNF‐α), which acts as an amplifier of inflammation in almost all acute inflammatory cascades, remained stable after intravenous injection of MelaBac cells, further suggesting the in vivo biosafety of MelaBac cells (Figure 5o).

### 2.5 Mechanistic Investigations by mRNA‐seq and Metabolic Analysis

**Figure 6:**
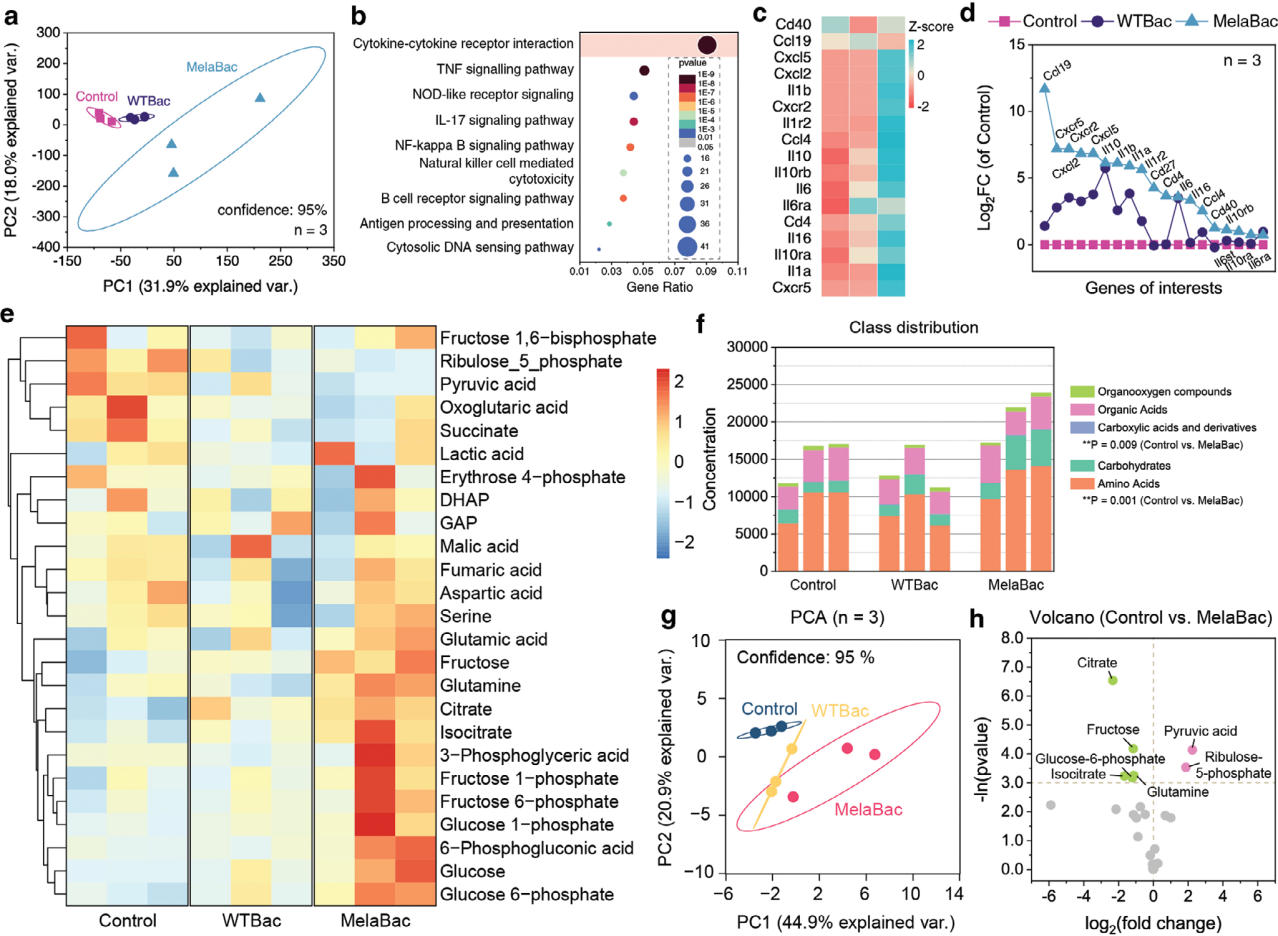
Mechanistic investigations by mRNA‐seq and metabolic analysis. a) Principal component analysis (PCA) of transcripts of tumor tissues of mice from different groups (control, WTBac, and MelaBac). Data are presented as they are, *n* = 3. b) Major KEGG enrichment pathways for DEGs between the MelaBac and Control groups. Data are presented as they are, *n* = 3. c,d) Heatmap c), and log_2_FC analysis d) of DEGs for samples in control, WTBac, and MelaBac groups. Data are presented as they are, *n* = 3. e,f) Heat map e), and class distribution f) of the metabolites quantified inside the tumor of mice from different groups. Data are presented as they are, *n* = 3. g) PCA of differentially quantified metabolites of mice from control, WTBac, and MelaBac groups. Data are presented as they are, *n* = 3. h, Volcano plot of differentially quantified metabolites of mice between control and MelaBac group. Data are presented as they are, *n* = 3.

To investigate the in‐depth key signaling pathways for MelaBac‐enabled tumor therapeutics, the xenografts of mice from different treatment groups were dissected and homogenized for RNA extraction and high throughput mRNA sequencing. We first initiated the principal component analysis (PCA) for all assayed 55 487 genes and found samples from the Control and WTBac groups allocated closely on the scale of two principal components, PC1 and PC2. In comparison, samples from the MelaBac group were distributed away from the other groups, with a non‐intersected confidence ellipse of 95% (**Figure** **6**a). We also identified that the differentially expressed genes (DEGs) were enriched to several signaling pathways, including cytokine‐cytokine receptor interaction, TNF signaling, Natural killer cell‐mediated cytotoxicity, NOD‐like receptor signaling, NF‐κB signaling, and Antigen processing and presentation pathway significantly, with higher significances to the enrichments between Control and WTBac groups (Figure 6b; Figure S26, Supporting Information). The highest enrichment score was allocated in the cytokine‐cytokine receptor interaction pathway, as revealed by the Gene Set Enrichment Analysis, preliminarily suggesting the prominent outburst of the cytokines with regulatory and cytotoxic functionalities (Figure S27, Supporting Information). We observe distinct expressions for immunity‐associated mRNA subsets such as CD40, CCL19, CXCL5, and other interleukins. Specifically, *CD40* mRNA was upregulated for tumor xenografts treated with MelaBac cells, mediating the activation of innate immunity and adaptive immunity.^[^ ^19^ ^]^ Chemokines and interleukins such as CXCL5, CXCR2, CXCL2, and CCL4 were all upregulated for xenografts treated with MelaBac cells rather than the Control and WTBac group, implicating the immune cell chemotaxis and antigen representation induced by the engineered MelaBac cells. Proinflammation‐associated interleukins such as IL‐1β, IL‐1α, and IL‐6 were also upregulated. Additionally, IL‐16 secretion, serving as an immune modulator, was upregulated to attract CD4^+^ T cells, monocytes, and eosinophils in the MelaBac group, validating the potent intratumoral immunostimulatory condition during tumor therapy (Figure 6c,d).^[^ ^20^ ^]^

Furthermore, 25 intratumoral metabolites were analyzed by targeted quantitative liquid chromatography‐tandem mass spectrometry (LC‐MS/MS) (Figure 6e). These metabolites could be classified into five classes (i.e., organooxygen compounds, organic acids, carboxylic acids and derivatives, carbohydrates, and amino acids). Among these metabolites, the total concentrations of amino acids, carboxylic acids, and carboxylic acid derivatives classes show a significant increase for the MelaBac group compared to the control group (Figure 6f). From PCA, distinct differences between the MelaBac and Control groups could be observed. In contrast, samples of the WTBac group were distributed spatially between the samples of the control and MelaBac groups (Figure 6g). We then further identify the significance of specific metabolites in comparing control and MelaBac groups. We found that pyruvic acid and Ribulose‐5‐phosphate were significantly downregulated from the MelaBac group, while citrate, fructose, glutamine, isocitrate, and glucose‐6‐phosphate were upregulated considerably (Figure 6h; Figure S28, Supporting Information). The downregulated metabolites implicate that the glycolysis flux has been blocked and shifted to a lower‐efficient gluconeogenetic pathway, thereby suppressing the localized tumor proliferation.^[^ ^21^ ^]^

### 2.6 In Vivo Chemical‐Induced CRC Therapeutics by MelaBac Cells and Anti‐Tumor Immunity Investigation

**Figure 7:**
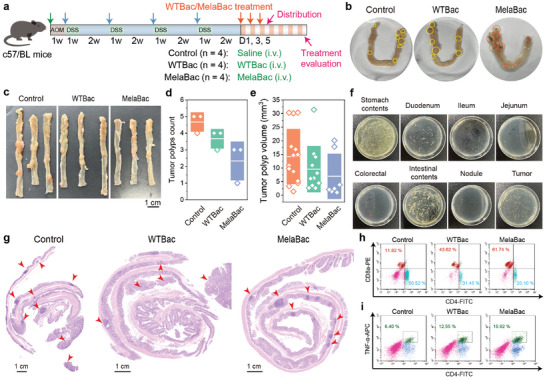
In vivo CRC tumor therapeutics by melabac cells and anti‐tumor immunity investigation. a) Schematic diagram of the establishment of murine models harboring chemical‐induced CRC and treatment schedule. b) Digital photographs of colon tissue after indicated treatments. c) Digital photographs of colon tissues of mice from control and different treatment groups at the end of the therapeutics. d,e) Quantification and statistical analysis of the d) counts and e) volumes of tumor polyps of mice from control and different treatment groups. Data are presented as they are. f) Digital photographs of MelaBac colonies in each segment of gastrointestinal tissue after MelaBac gavage three times. g) Representative histological microscopic images of the colon tissues (presented as Swiss rolls) of mice from control and different treatment groups. h,i) Flow cytometric results of immune cells including h) CD4^+^ and CD8^+^ T cells and i) CD4^+^TNFα^+^ T cells of spleen tissues.

Chemical‐induced CRC features with the onset of multiple polyps spread on the mucous membranes, making it challenging for therapeutics based on traditional treatment modalities.^[^ ^22^ ^]^ With the native tumor tropism of MelaBac, selective accumulation and therapeutics enabled by MelaBac cells is highly promising. To validate the hypothesis, murine CRC models were established on C57BL/6 mice with azoxymethane (AOM) intraperitoneal administration and dextran sulfate sodium (DSS) salt‐containing water drinking (**Figure** **7**a). According to the treatment schedule, twelve mice with CRC were allocated into three groups: Control (Saline), WTBac group (oral administration of WTBac every other day for three times), and MelaBac group (oral administration of MelaBac every other day for three times). After oral administration of bacteria on day 6, one mouse in each group was euthanized, and the whole colon tissues (from stomach to anus) were dissected. Compared to the control group, colon polyps of mice from the MelaBac group were pigmentated by MelaBac cells, which could be easily observed by the naked eye (Figure 7b), providing potential applications for intraoperative guidance of tumor determination. At the end of the therapeutic timeframe of 14 days, all mice were euthanized, and their colon tissues were dissected for tumor polyp evaluation (Figure 7c). Sporadic CRC polyps were pigmentated from the colon of mice from the MelaBac group, indicating the tumor foci. The total number of tumor polyps for these groups was recorded as 14, 11, and 7, respectively. MelaBac cell therapeutics induce a substantial decline in the polyp number (Figure 7d). For the average dimension of the tumor polyps, WTBac cells or MelaBac cells therapeutics restricted the average polyp volume to 14.20 and 9.60 mm^3^ compared to 6.96 mm^3^ in the Control group (Figure 7e). We further isolated the stomach, duodenum, jejunum, colorectal, intestine, nodule, and polyp, homogenized and inoculated on the plates with ampicillin selection. We found MelaBac (with ampicillin resistance) bacterial colonies mainly grow onto the plates of the stomach, intestinal, and polyp sites. The results demonstrate the effective accumulation and targeting performance of MelaBac inside the tumor foci through oral administration. We could also observe that MelaBac cells were not distributed in intestinal nodules, which could be due to the absence of microvessels within benign nodules (Figure 7f). We also inspected the H&E‐stained colon section for tumor polyp identification and indication. We found that MelaBac cells exhibit the most outperformed therapeutic effect in the tumor polyp counts and dimensions (Figure 7g).

To investigate antitumoral immunity further, the spleen populations of specific immune cells (CD4^+^ T cells, CD8^+^ T cells) were quantified and evaluated. Mice after varied treatments were observed with gradually decreased CD4^+^ T percentage and increased CD8^+^ T percentage (Figure 7h). MelaBac treatment led to the highest increase of CD8^+^ T cells and TNFα^+^ T cells to be 68.12% and 16.92% respectively (Figure 7i; Figure S29, Supporting Information). Activated CD69^+^CD8^+^ T cells were identified to increase from 42.31% to 55.03% significantly (Figure S30, Supporting Information), collectively confirming the robust activation of prominent antitumoral adaptive immunity for tumor destruction.

## 3 Conclusion

In summary, we have designed, screened, and constructed the tyrosinase‐expressing *E. coli* MG1655 bacterial cells with optimized melanin generation performance, designated as MelaBac cells. With p2800 as the constitutive promoter, the MelaBac cells could produce 393.5 U of melanin per 10^7^ c. f. u. cells within 24 h in the presence of copper ions and tyrosine substrate, leading to the prominent pigmentation effect. The pigmentated MelaBac cells enable the NIR‐initiated photonic hyperthermia therapeutic modality, effectively eradicating the murine CT26 colon tumor cells. During in vivo therapeutics, we found that intravenous administration of MelaBac cells could induce apparent pigmentation of the xenografts in 3 days. Through 808 nm laser irradiation, complete eradication against the tumor xenograft could be demonstrated. For AOM/DSS‐induced CRC, sporadic pigmentation of the multiple tumor polyps is also indicated. These tumor polyps were substantially destroyed based on the tumor‐targeted immunotherapy initiated by the anti‐tumor immunity activation by MelaBac cells. Collectively, we have innovated a tumor‐targeted intratumoral pigmentation strategy that could facilitate photonic hyperthermia and immunotherapeutic interventions with high selectivity and biocompatibility, providing naked eye visualization‐based intraoperative navigation promises and intelligent living immunotherapeutic based on genetically engineered bacterial cells.

Naked eye visualization‐based intraoperative navigation has provided convenient sights to determine the boundary of tumor for surgery removal, without the necessity of complicated facilities and technical demands. Our findings confirm that the MelaBac cells could effectively and selectively accumulate inside the tumor region for tumor‐specific pigmentation. We have elaborated these experiments on both subcutaneous tumor xenograft model and chemical‐induced CRC model. We found that MelaBac cells could pigmentate the subcutaneous tumor xenografts much more effectively than the chemical‐induced CRC tumor polyps. For subcutaneous tumor xenograft, an average tumor volume of 100 mm^3^ could retain higher dose of MelaBac cells for pigmentation. For sporadic CRC tumor polyps, the polyp volume is rarely larger than 30 mm^3^ in murine model. The differences are possibly due to the accumulated amount of MelaBac cells inside varied types of tumors. Subcutaneous xenografts are larger in tumor volume and actively supported with sufficient blood supply, while AOM/DSS‐induced adenomas with small volumes are poor in blood supply. Though with limitations of tumors with larger size and better blood supply, the present pigmentation strategy still preserves great clinical potential for tumor visualization in human beings with larger dimensions of tumor. In addition, the pigmentation performance deserves further optimization in order to achieve a lower visual detection limit. Investigations on the pigmentation in other types of large‐size tumors, such as hepatocellular carcinoma, are also appealing to help identify the boundary for clinical convenience during surgical resection.

*Statistical analysis*: Data are presented as mean ± SD or as they are. Sample sizes are presented in the figure legend. Data significances were analyzed by a two‐tailed paired student's *t*‐test. ^*^ *p* < 0.05, ^**^ *p* < 0.01, ^***^ *p* < 0.001, and *p* > 0.05 for non‐significant. Origin 2021 was used for statistical analysis.

## Conflict of Interest

The authors declare no conflict of interest.

## Supporting information

Supporting Information

## Data Availability

The data that support the findings of this study are available in the supplementary material of this article.
