# Supplementary material for: Genetically Designed Living Bacteria with Melanogenesis for Tumor‐Specific Pigmentation and Therapeutic Intervention
Source: Adv Sci (Weinh). 2024 Jun 18;11(31):2402709. doi: 10.1002/advs.202402709 (PMC11336949; doi:10.1002/advs.202402709)
Supplement: Supplementary file 1 — Supporting Information [file ADVS-11-2402709-s001.docx]

Supporting Information

Genetically Designed Living Bacteria with Melanogenesis for Tumor-Specific Pigmentation and Therapeutic Intervention

Liying Wang,^1^ Qi Wu,^1^ Qi Lyu,^2^ Dan Lu,^2^ Lehang Guo,^1^ Chao Zhong,^3^ Min Wang,^4^ Chang Liu,^1^* Bolin An,^3^* Huixiong Xu,^2^* and Minfeng Huo^1^*

**1. Experimental Section/Methods**

**Materials.** 1640 Medium and fetal bovine serum (FBS) were obtained from Gibco (USA). DCFH-DA, Calcein-AM/PI, CCK-8, and Annexin V-FITC/PI staining kits were purchased from DOJINDO. Co.. GM-CSF, IL-4, collagen IV, and DNase I were purchased from Beyotime Biotechnology. The LB broth and cell culturing reagents were purchased from Sangon Biotech Co., Ltd.. TNF-α, IL-6, and IL-1β ELISA kits were purchased from Beyotime Biotechnology. Ampicillin, l-Tyrosine, tyrosinase from mushroom (Cat#: T128536) and CuSO_4_ were purchased from Aladdin Biochemical Co., Ltd.. His tag antibodies were purchased from Transgen Biotech. Ghost dye 780 (Cat#: 13-0865) was purchased from TUNBO Reagent. CD45-VF450 (Cat#: Ab241940) was purchased from abcam. CD3e-Percp-cy5.5 (Cat#: 551163), CD4-FITC (Cat#: 553651), CD8a-BV786 (Cat#: 563332), CD8a-PE (Cat#: 553033), CD11c-FITC (Cat#: 568943), CD86-PE (Cat#: 561963) and CD86-PE-cy7 (Cat#: 560582), CD86-APC (Cat#: 558703) and CD206-PE (Cat#: 568273) were purchased from BD BioSciences. CD80-APC (Cat#: 104740) was purchased from Biolegend. TNF-α-APC (Cat#: 17-7349-82) were purchased from Thermo Fisher Invitrogen. Validation and references of the application of these antibodies were on corresponding category pages of the supplier.

**Characterizations.** Transmission Electron Microscopic (TEM) images were recorded on a JEOL-JEM-2100F electron microscope. Scanning Electron Microscopic (SEM) images were recorded on Sigma 300. UV-vis spectra were recorded on Shimadzu UV-3101PC. Ultrasound images were recorded on Mindray Resona R9. Photoacoustic images were obtained from VEVO LAZR X produced by Fujifilm VisualSonics.

**Strain construction.** TyrA and Cofactor sequences were amplified and cloned to the AmpR selective plasmid with the pJ23108, p1400, or p2800 promoter. The plasmid was then transformed into the competent *E. coli* MG1655 bacterial strain (cat# BTN12-211y from Beijing BaiAoLaiBo Science and Technology Co. Ltd.) to construct the bacterial strain. LB agar plates with ampicillin (50 μg/ml) were used to screen the bacterial cells with transformed plasmids. All cloning followed standard molecular cloning protocols and was verified by DNA sequencing.

**SEM observation.** WTBac cells or MelaBac cells were supplemented into 10 ml of LB with or without the addition of ampicillin (50 μg/ml), CuSO_4_ (40 μg/ml), and l-tyrosine (0.5 mg/ml) for overnight incubation. These bacterial cells were then collected by centrifugation (7000 rpm, 10 min) and freeze-dried into powder for morphology observation by SEM.

**Western blot analysis.** MelaBac cells with different promoters (2×10^9^ c.f.u.) were supplemented in 1 mL LB, treated with lysosome (1 mL, 1 mg/mL) under 37 ^o^C for 30 min, and redispersed in 0.5 mL cold RIPA for 10 min. The cells were then centrifuged at 7000 rpm for 10 mins at 4 ^o^C to collect the supernatant for western blot analysis using mouse anti-Histag monoclonal antibody.

**Kinetic evaluation of melanogenesis.** MelaBac cells with different promoters were dispersed in 1 mL LB medium (10^9^ c.f.u.) containing ampicillin, CuSO_4_ (0.2 and 0.4 mg) and l-Tyrosine (0.5, 1 and 2 mg). Upon initiation in the 48-well plate, the optical absorptions of these assays at 600 nm were recorded in the microplate reader every 30 min. The plate was photographed for comparison at predetermined timepoints (2, 4, 6, 8 and 12 h). For the melanogenesis of EcMG^TYR^-p2800, the conditions were further optimized to the following: CuSO_4_ (0.2, 0.1, 0.05 mg), l-Tyrosine (0.5, 1 and 2 mg) and time (2, 4, 6, 8, 12 and 24 h).

**Cell culture.** Murine CT26 colon tumor cell line (Cat#: TCM37) and murine RAW264.7 macrophage cell line (Cat#: TCM13) were obtained from the Cell Bank, the Committee of Type Culture Collection of the Chinese Academy of Sciences. These cells have not been listed in the cross-contaminated or misidentified cell lines (v8.0, 2016) and are qualified based on cell line tests, including morphology identification, isoenzymes, and mycoplasma. CT26 cells were cultured in 1640 medium with 10% fetal bovine (FBS) and 1% Penicillin-Streptomycin at 37 ^o^C in a humidified atmosphere with 5% CO_2_. RAW264.7 cells were cultured in DMEM with 10% fetal bovine (FBS) and 1% Penicillin-Streptomycin at 37 ^o^C in a humidified atmosphere with 5% CO_2_.

***In vitro cell viability assay.*** MelaBac (1×10^8^ c. f. u.) were added into the 24-well plate with pre-inoculated CT26 cells (1×10^5^ cells per well) and cultured in 1640 medium supplemented with CuSO_4_ (0, 50 or 100 per well) and l-tyrosine (0, 0.5 or 1 mg per well) without Penicillin- Streptomycin. For wells assigned to the NIR irradiation group, cells were irradiated with 808 nm laser (1 W/cm^2^, 8 min) in 12 h or 24 h. All the wells were further assayed by cell counting kit-8 to evaluate the cell viability.

***Confocal observation.*** CT26 cells were co-incubated with MelaBac cells for cell viability visualization with indicated treatments. These cells were stained with Calcein-AM/PI dyes for 15 min before confocal microscopic observation (Olympus FV1000).

***Flow cytometry.*** According to an established protocol, bone-marrow-derived DCs were obtained from the 5-week-old female C57BL/6 mice. These DCs were seeded in the lower chamber of a 6-well transwell setup (5×10^5^ cells per well) and cultured overnight. Bacterial cells or tumor cells with different treatments were collected and placed into the upper chamber of the transwell setup. After co-incubation for 12 h, DCs were collected and stained with CD11c-FITC, CD80-APC, and CD86-PE antibodies for flow cytometric analysis on Beckman Cytoflex. During flow cytometry analysis, 1×10^5^ cells were collected. Supernatants were also collected for ELISA assays, including TNF-α, IL-1β, IL-12p70, IFN-γ, and IL-6. RAW264.7 cells with indicated treatments were stained with CD86-APC and CD206-PE for further flow cytometric measurements.

**Animals.** All mice (including Balb/c nude mice, Balb/c, and C57BL/6 mice) were purchased from Beijing Charles River Experimental Animal Technology Co., Ltd. and housed in specific-pathogen-free facilities with free access to food and water. All the *in vivo* animal experiments were performed under the Laboratory Animal Ethics Committee guidelines of Shanghai Tenth People’s Hospital (SHDSYY-2023-6600).

***In vivo* tumor therapeutics against xenograft-bearing nude mice.** 4-weeks-old Balb/c nude mice were subcutaneously injected with CT26 tumor cells (1×10^6^ cells in 100 μl per mouse). The xenografts were allowed to grow to 150 mm^3^ and mice were divided into 6 groups randomly: Control (i.v. injection of saline); WTBac (i.v. injection of 1×10^8^ c. f. u. per mouse), MelaBac (i.v. injection of 1×10^8^ c. f. u. per mouse), WTBac + NIR (1×10^8^ c. f. u. per mouse + 808 nm laser (24 h post bacterial administration, 1 W/cm^2^, 8 min)), MelaBac + NIR (1×10^8^ c. f. u. per mouse + 808 nm laser (24 h post bacterial administration, 808 nm laser: 1 W/cm^2^, 8 min)). During the evaluation timeframe of 11 days, the body weight of mice was recorded every day. The xenograft dimensions were measured using a digital calliper every day. At the end of the treatment and evaluation, all mice were sacrificed by painless cervical dislocation according to the guidelines.

***In vivo* evaluation of hemodynamics in xenograft-bearing murine models.** 4-weeks-old Balb/c nude mice were subcutaneously injected with CT26 tumor cells (1×10^6^ cells in 100 μl per mouse). The xenografts were allowed to grow to 300 mm^3^ and mice were divided into 3 groups randomly: Control (i.v. injection of saline); WTBac (i.v. injection of 1×10^8^ c. f. u. per mouse), and MelaBac (i.v. injection of 1×10^8^ c. f. u. per mouse). In 24 h post administration of bacterial cells, mice were anesthetized by pentobarbital for ultrasound inspections (Mindray R9). Sonovue was administered intravenously before the ultrasound inspections using Mindray R9 to visualize the vasculature. Ultrasound elastography inspections were conducted using SuperSonic Imagine (Aix-en-Provence, France). Photoacoustic images and O_2_-saturation modes were obtained on VEVO LAZR-X.

***In vivo* tumor therapeutics against xenograft-bearing Balb/c mice.** 4-weeks-old Balb/c mice were subcutaneously injected with CT26 tumor cells (1×10^6^ cells in 100 μl per mouse). The xenografts were allowed to grow to 150 mm^3^ and mice were divided into 6 groups randomly: Control (i.v. injection of saline); WTBac (i.v. injection of 1×10^8^ c. f. u. per mouse), MelaBac (i.v. injection of 1×10^8^ c. f. u. per mouse), WTBac + NIR (1×10^8^ c. f. u. per mouse + 808 nm laser (24 h post bacterial administration, 1 W/cm^2^, 8 min)), MelaBac + NIR (1×10^8^ c. f. u. per mouse + 808 nm laser (24 h post bacterial administration, 808 nm laser: 1 W/cm^2^, 8 min)). One mouse from each group was euthanized for xenograft dissection and photographing the other day of the NIR therapeutics. During the evaluation timeframe of 15 days, the bodyweight of mice was recorded every other day. The xenograft dimensions were measured using the digital calliper every other day. At the end of the treatment and evaluation, all mice were sacrificed by painless cervical dislocation according to the guidelines. The xenografts of the mice from different groups were dissected for weighting and tumor volume measuring. These tumor xenografts were further sliced into ultrathin sections for FISH immunofluorescence identification, HIF-1α immunohistochemical inspections, H&E staining, and TUNEL staining, respectively.

***In vivo* biosafety and hematic evaluation.** Four-weeks-old female Balb/c mice were intravenously injected with MelaBac cells (1×10^9^ c. f. u. per mouse), followed by whole blood and serum collection at predetermined timepoints (0, 2, 6, 12, 24, 48, and 72 h). Whole blood was used for blood routine analysis. Serum was used for ELISA assays, including IL-1β, IL-6, and TNF-α.

*In vivo* tissue targeting evaluation. Four-weeks-old female Balb/c mice were intravenously injected with MelaBac cells (1×10^9^ c. f. u. per mouse), followed by major organ collection at predetermined timepoints (0, 2, 6, 12, 24, and 48 h). Major organs were dissected, frozen with liquid nitrogen, homogenized, and plated into the LB agar plates for remaining bacterial counting.

**mRNA-seq.** 4-weeks-old Balb/c mice were subcutaneously injected with CT26 tumor cells (1×10^6^ cells in 100 μl per mouse). The xenografts were allowed to grow to 150 mm^3^ and mice were divided into 3 groups randomly: Control (i.v. injection of saline), WTBac (i.v. injection of 1×10^8^ c. f. u. per mouse), and MelaBac (i.v. injection of 1×10^8^ c. f. u. per mouse). In 2 days post-administration of the bacterial cells, mice were sacrificed by painless cervical dislocation according to the guidelines. The xenografts of the mice from different groups were dissected and frozen in liquid nitrogen.

Total RNA was isolated using a TRIzol total RNA extraction kit (TIANGEN, Cat.No. DP424), which yielded > 2 μg of total RNA per sample. RNA quality was examined by 0.8 % agarose gel electrophoresis and spectrophotometry. High-quality RNA with a 260/280 absorbance ratio of 1.8-2.2 was used for library construction and sequencing. Illumina library construction was performed according to the manufacturer's instructions (Illumina, USA). Oligo-dT primers are used to transverse mRNA to obtain cDNA (APExBIO, Cat. No. K1159). Amplify cDNA for the synthesis of the second chain of cDNA. Purify cDNA products by AMPure XP system (Beckman Coulter, Beverly, USA). After library construction, library fragments were enriched by PCR amplification and selected according to a fragment size of 350-550 bp. The library was quality-assessed using an Agilent 2100 Bioanalyzer (Agilent, USA). The library was sequenced using the Illumina NovaSeq 6000 sequencing platform (Paired end150) to generate raw reads.

Raw paired-end fastq reads were filtered by TrimGalore to discard the adapters and low-quality bases via calling the Cutadapt tool.^[1]^ The clean reads obtained were then aligned to the mm10 mouse genome using HISAT2 ^[2]^, followed by reference genome-guided transcriptome assembly and gene expression quantification using StringTie.^[3]^ Differentially expressed genes (DEGs) were identified by DEseq2 ^[4]^ with a cut-off value of log2|fold-change|>1 and p-adjust <0.05. The clusterProfiler ^[5]^ was used to perform functional enrichment analysis for the annotated significant DEGs, the potential genes in identified modules based on gene ontology (GO), and KEGG pathway categories. Terms with pvalue<0.05 were considered significant. Gene set enrichment analysis (GSEA) was performed by the function in package clusterProfiler with a gene list sorted by log2 fold-change.

**Metabolites quantifications.** The frozen xenografts were thawed on an ice bath to reduce sample degradation. 10 mg xenograft was weighed into a 1.5 mL EP tube, and the sample was supplemented with 20 μL ultrapure water and 10 magnetic beads, homogenized for 3 mins (BB24, Next Advance, Inc., Averill Park, NY, USA), and then supplemented with 120 μL pre-cooled methanol solution containing internal standard and homogenized again for 3 min. The sample was further centrifuged at 18000 g for 20 min at 4°C (Microfuge 20R, Beckman Coulter, Inc., Indianapolis, IN, USA). 100 μL of the supernatant was aliquoted in a 1.5 mL EP tube and concentrated at 4°C (Labconco, Kansas City, MO, USA), supplemented with 100 μL 80 % methanol solution for reconstitution, centrifuged, and transferred to 96-well plate. For sample injection analysis, the ultra-high pressure liquid chromatography-triple quadrupole mass spectrometer (ACQUITY-I UPLC / Xevo TQ-S) from Waters Corporation was employed to detect the indicated metabolites. The instrument performs system optimization and maintenance every 48 hours.

***In vivo* chemical-induced CRC establishment.** To establish the murine models with CRC, 4-week-old C57BL/6 mice were intraperitoneally administrated with azoxymethane (AOM) on Day 1. On day 7, mice were supplemented with dextran sulphate sodium (DSS) salt containing drinking water for 1 week. Then DSS was removed from the drinking water for 2 weeks. The DSS drinking-resting cycle was repeated four times to establish the CRC model. These mice were divided into 3 groups randomly: Control (i.v. injection of saline, injection on Day 1, 3, 5); WTBac (i.v. injection of 1×10^8^ c. f. u. per mouse, injection on Day 1, 3, 5) and MelaBac (i.v. injection of 1×10^8^ c. f. u. per mouse, injection on Day 1, 3, 5). On day 6, one mouse from each group was euthanized for colon dissection and photographing. The colon's stomach, duodenum, ileum, jejunum, colorectal, intestinal, nodule, and tumor polyp were separated, homogenated, and inoculated onto the selective LB agar plates for the remaining bacterial culture. On day 14, all mice were sacrificed by painless cervical dislocation according to the guidelines. The colon tissue of mice from different groups was dissected and photographed. The counts and dimensions of the tumor polyps on the colon were recorded. The colon tissue was then scrolled to a Swiss-roll-like pattern for fixation and ultrathin section preparation. Further H&E staining of the colon tissue was performed.

**2. Supplementary Figures**


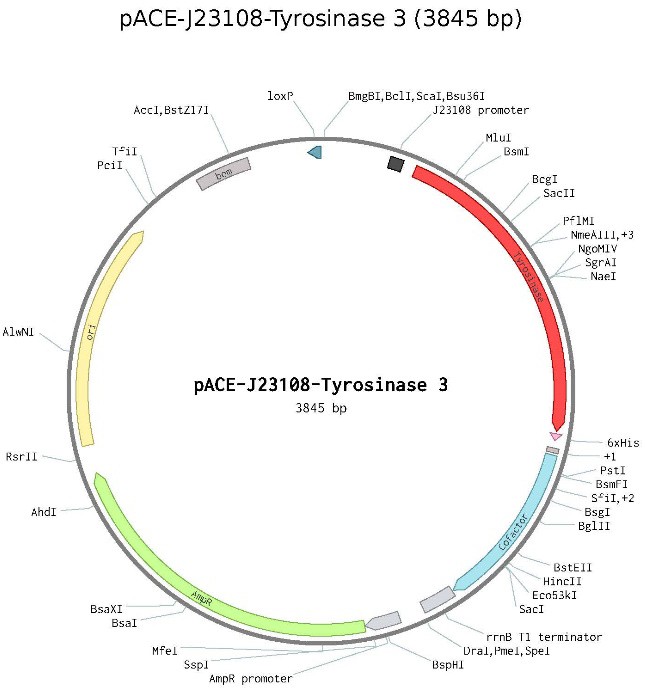


**Figure S1.** Sequence map of pACE-pJ23108-Tyrosinase.


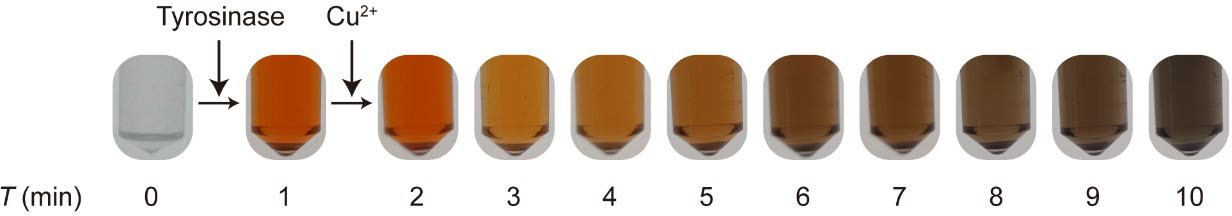


# Figure S2. Digital photographs of the chromogenic kinetics in 10 min of the solution containing l-tyrosine, tyrosinase, and Cu^2+^.


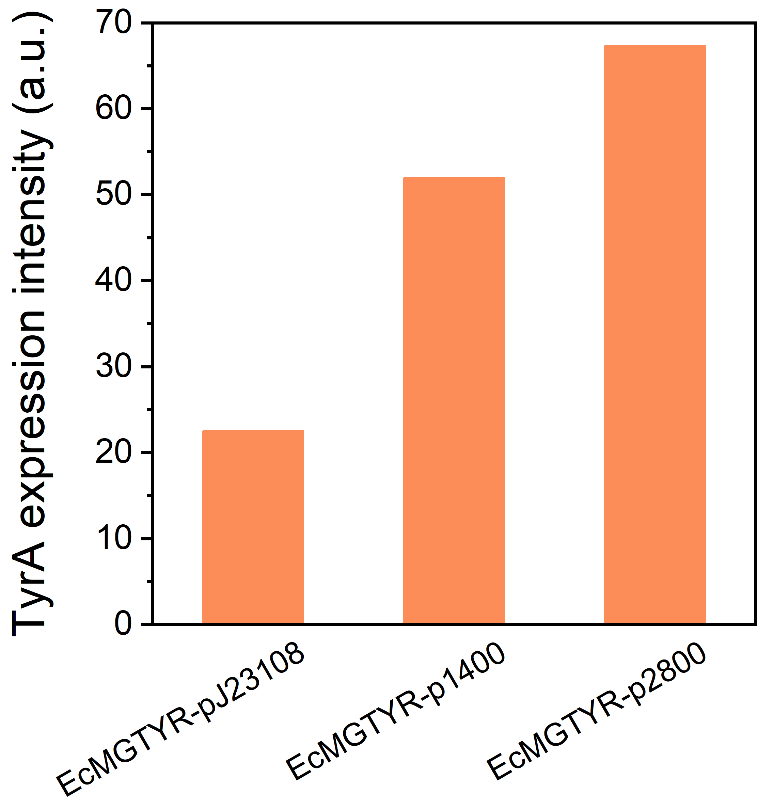


**Figure S3**. WB quantification of TyrA expression for EcMG^TYR^-pJ23108, EcMG^TYR^-p1400 and EcMG^TYR^-p2800.


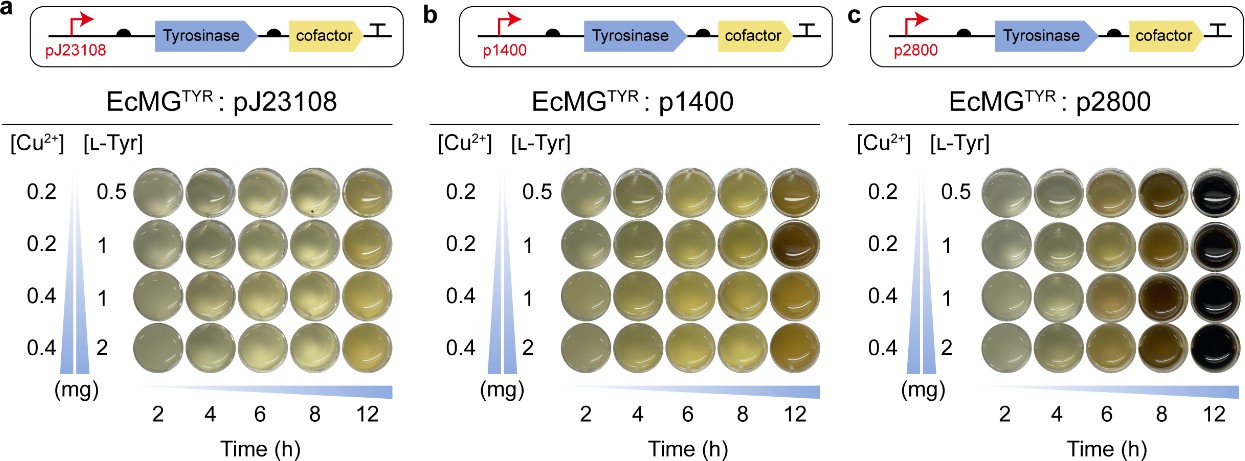


# Figure S4. Melanogenesis activity for EcMG^TYR^-pJ23108, EcMG^TYR^-p1400 and EcMG^TYR^-p2800 with varied concentrations of Cu^2+^ and l-tyrosine along time.


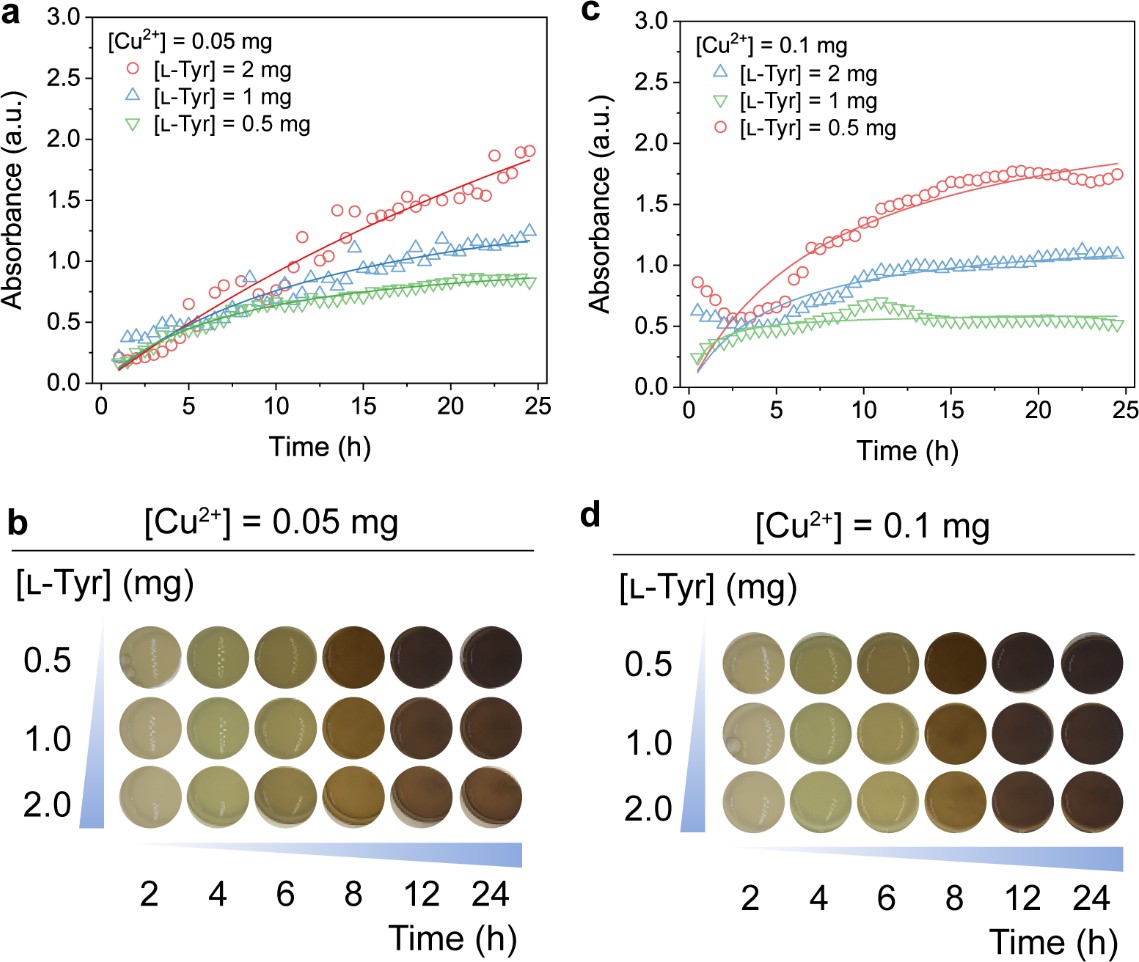


# Figure S5. Time-course optical absorbance (a, c) and corresponding digital photographs (b, d) of LB medium containing EcMG^TYR^-p2800 (10^8^ c. f. u.) in the presence of different concentrations of L-tyrosine (0.5, 1 and 2 mg) and copper ions (0.05 and 0.1 mg) along time (2, 4, 6, 8, 12 and 24 h).


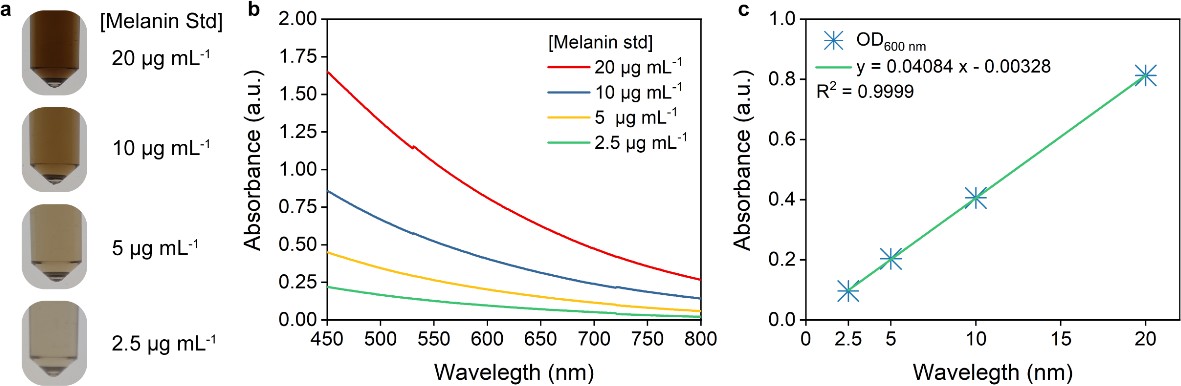


# Figure S6. Digital photographs (a), UV-vis optical absorptions (b) of the solution containing different concentrations of melanin standard: 20 μg/mL, 10 μg/mL, 5 μg/mL, and 2.5 μg/mL. c, Standard curve for melanin standard as calibrated at OD600 nm.


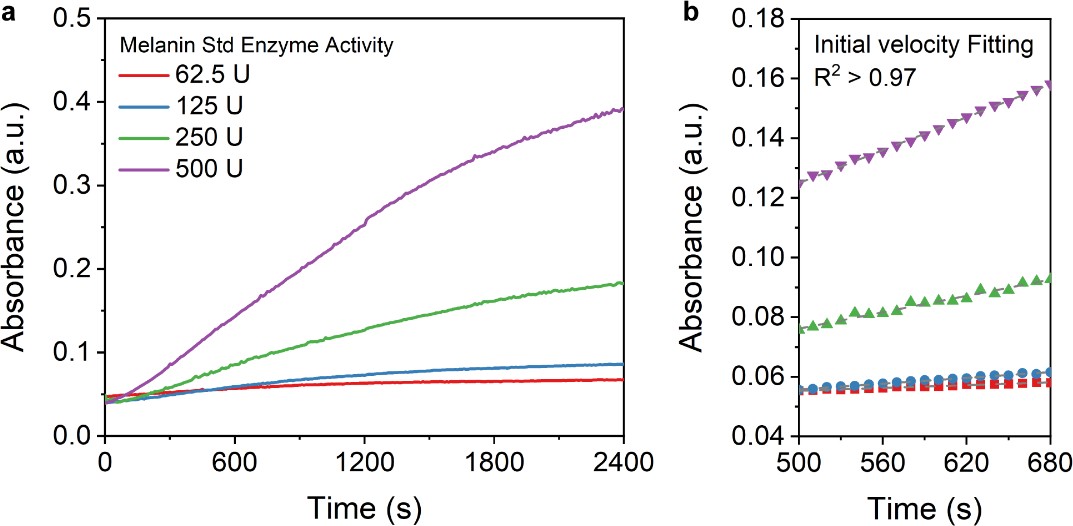


# Figure S7. a, Melanogenesis chromogenic curve of the solution containing tyrosinase standard: 62.5 U, 125 U, 250 U, and 500 U. b, Initial velocity curve and corresponding fitting of the chromogenic curves.


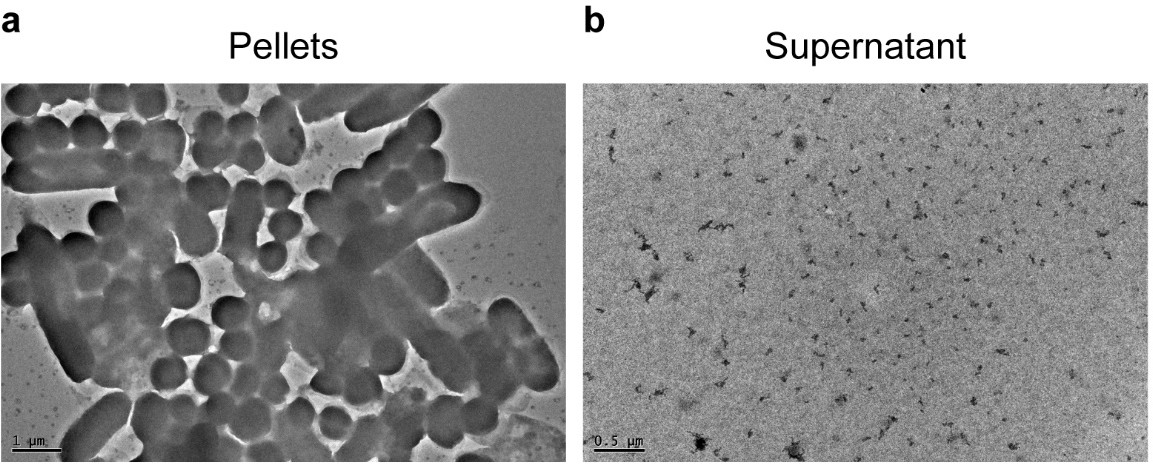


# Figure S8. TEM images of the pellets (a) and supernatant (b) of the pigmentated MelaBac cells after centrifugations.


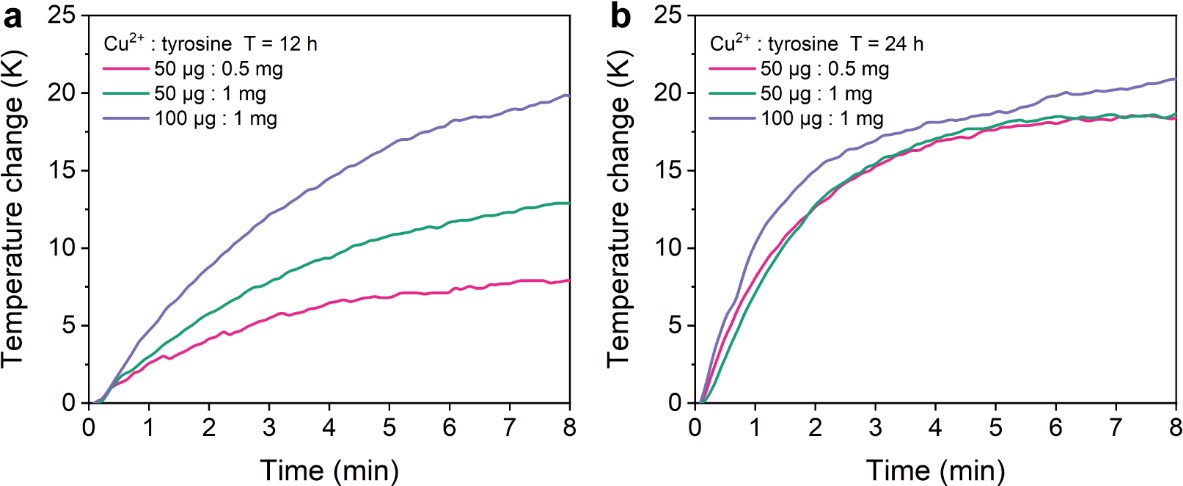


# Figure S9. a, b, Temperature elevation curve of cellular medium under 808 nm laser irradiation after coincubation with MelaBac cells, Cu^2+^ and l-tyrosine for 12 h and 24 h.


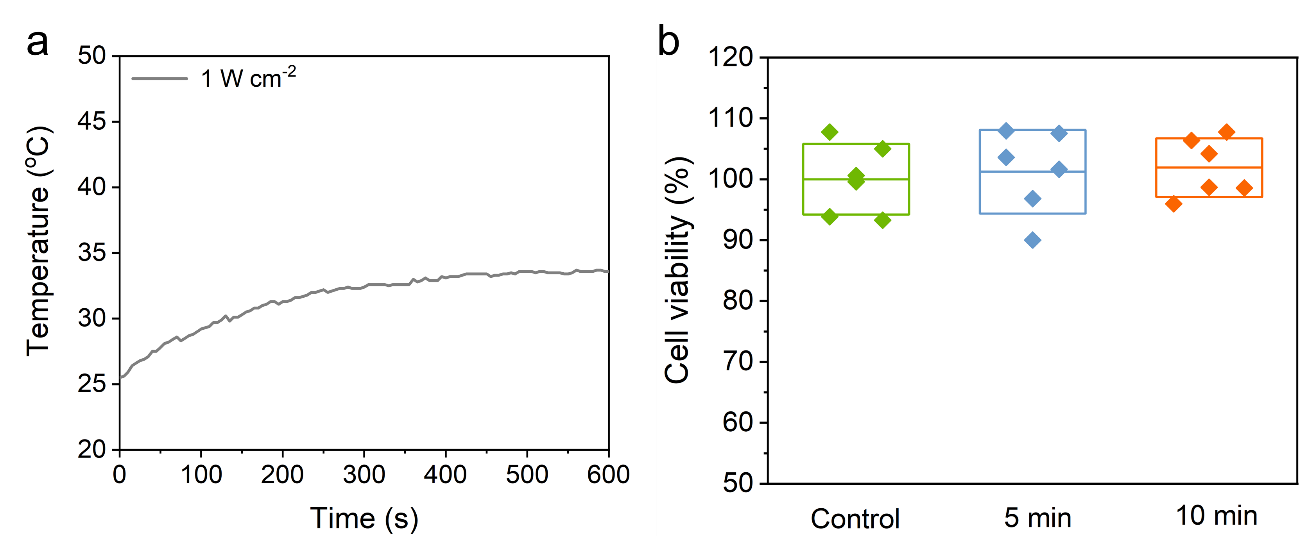


**Figure S10.** a-b, Temperature elevation curve of cellular medium under 808 nm laser irradiation with power density of 1 W/cm^2^ (a) and corresponding cell viability after irradiation for 5 min and 10 min (b).


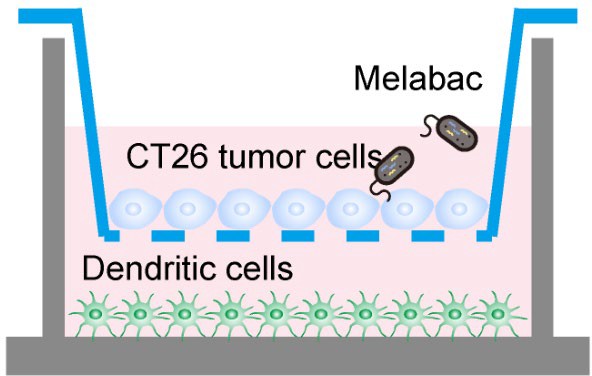


# Figure S11. Transwell design for DC maturation evaluation.


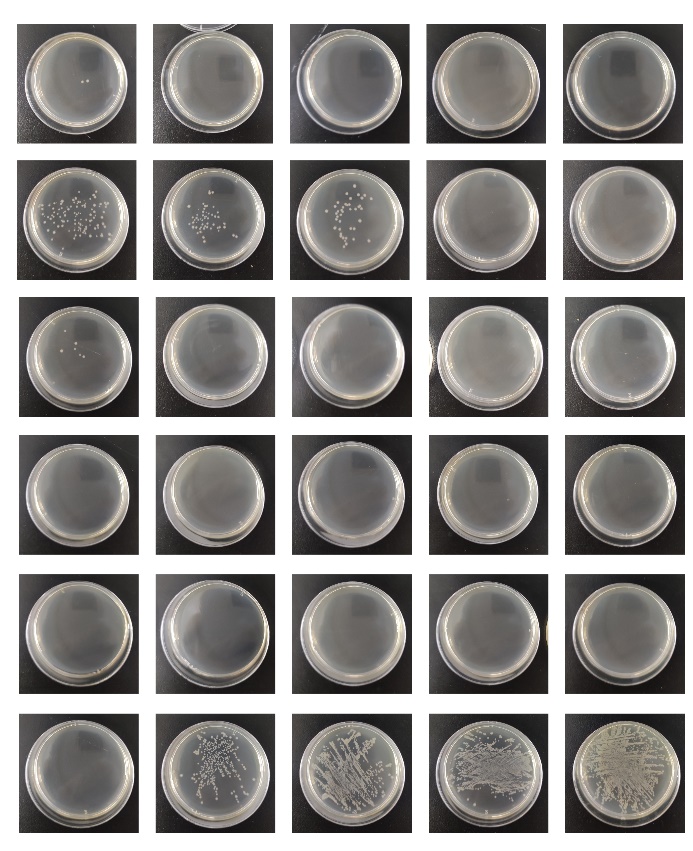


# Figure S12. Representative images of ampicillin-added LB-agar plates inoculated with tissue grinding fluid including heart, liver, spleen, lung, kidney, and tumor at different time points after MelaBac injection (2, 6, 12, 24, and 48 h).


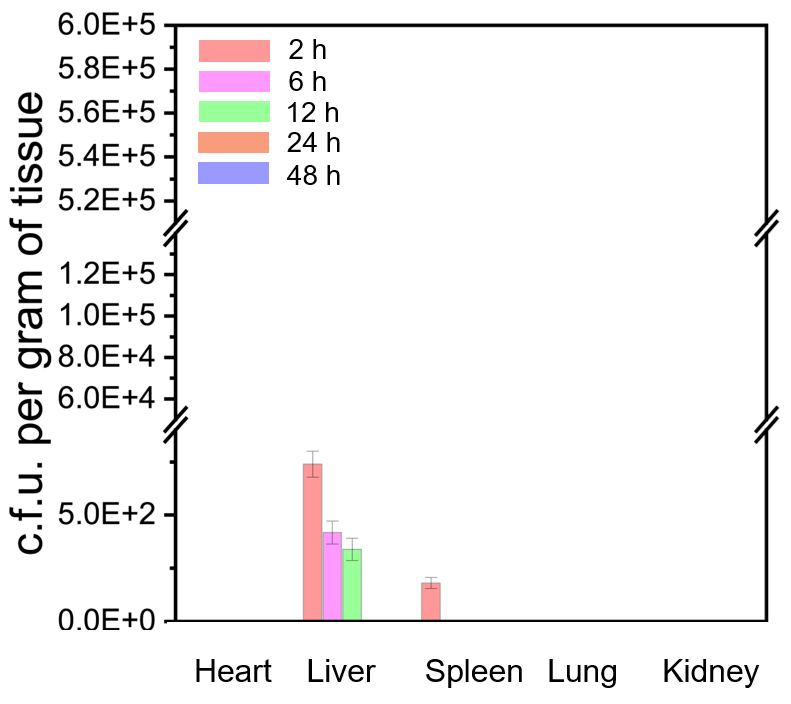


**Figure S13.** Counting results of MelaBac within tissues of heart, liver, spleen, lung and kidney at time points of 2 h, 6 h, 12 h, 24 h and 48 h.


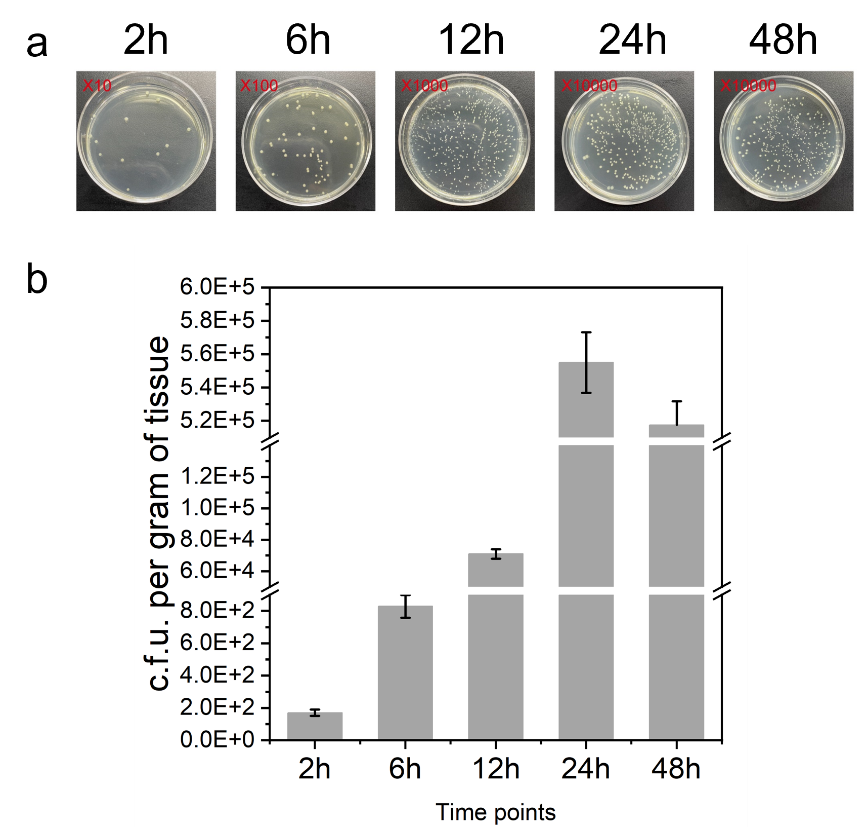


**Figure S14.** a-b, Digital photographs of the plating result (a) and of corresponding colony counting (b) of MelaBac within tumor tissues after dilution at time points of 2 h, 6 h, 12 h, 24 h and 48 h.


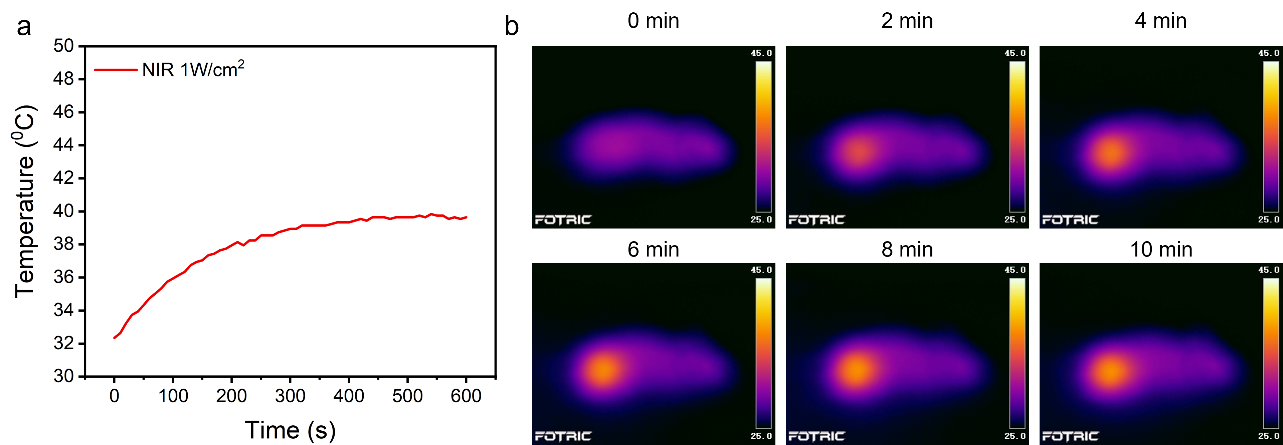


# Figure S15. a-b, Temperature elevation curve (a) and corresponding infrared imaging photographs (b) of untreated mice irradiated by 808 nm laser (1 W/cm^2^, 8 min).


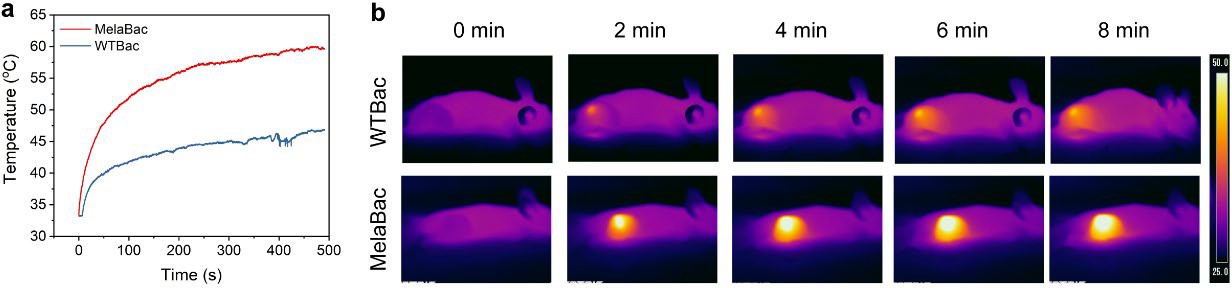


# Figure S16. a-b, Temperature elevation curve (a) and corresponding infrared imaging photographs (b) of mice irradiated by 808 nm laser (1 W/cm^2^, 8 min) in 24 h post-WTBac or MelaBac injection.

#
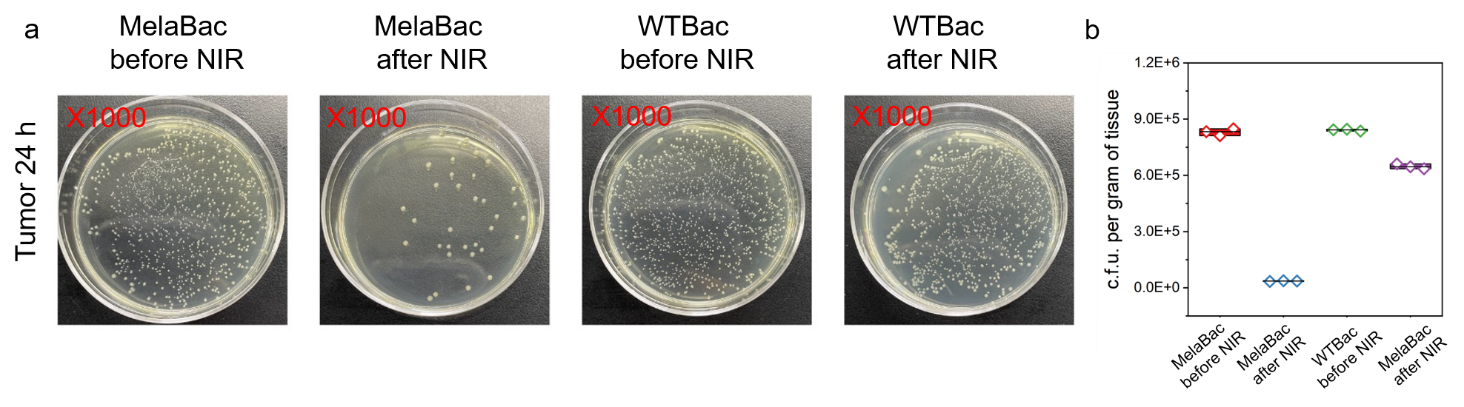


# Figure S17. a-b, Digital photographs (a) and counting results (b) of MelaBac and WTBac within tumor tissues before and after NIR treatment.

#
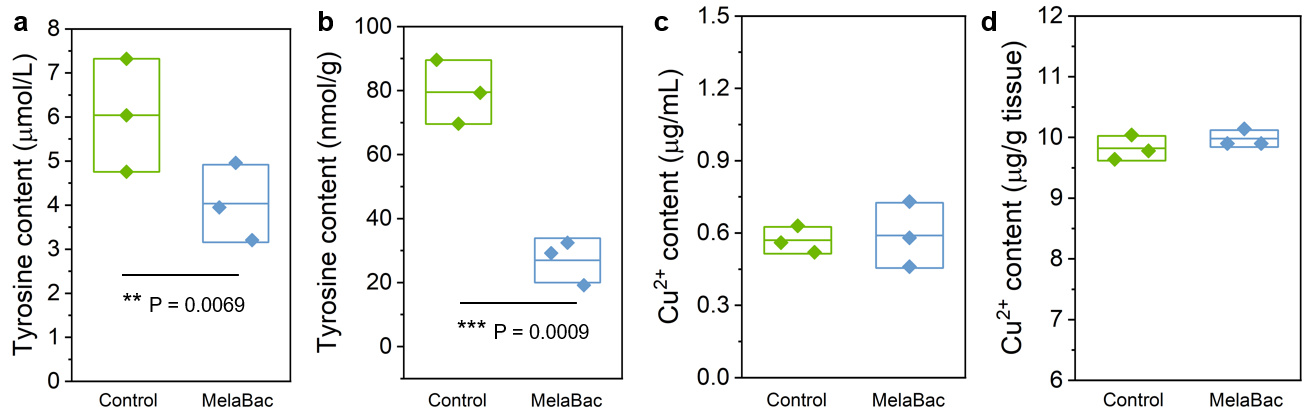


# Figure S18. a-b, Tyrosine content analysis of the serum (a) and tumor tissues (b) of mice from control group and MelaBac group in 24 h post bacteria injection. c-d, Copper content analysis of the serum (c) and tumor tissues (d) of mice from control group and MelaBac group in 24 h post bacteria injection.

#
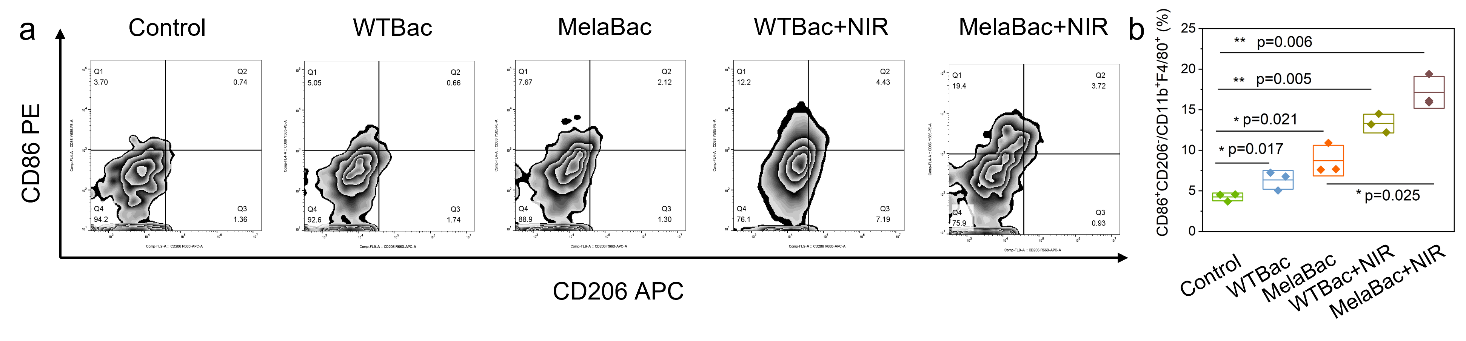


# Figure S19. Representative flow cytometry images of macrophages isolated from tumor tissues in mice from each group and corresponding statistical analysis of M1 phenotype.


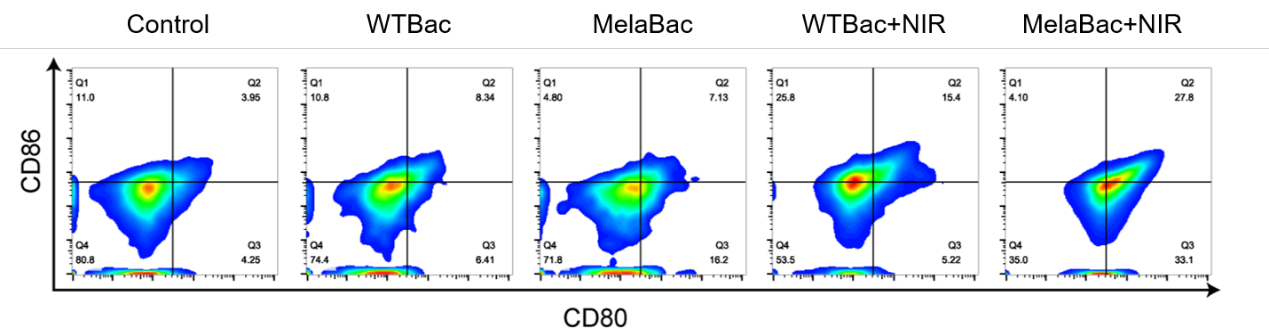


# Figure S20. Flow cytometry images of DCs extracted from lymph nodes of mice from different groups stained with CD11c-FITC/CD80-APC/CD86-PE-Cy7 antibodies.

#
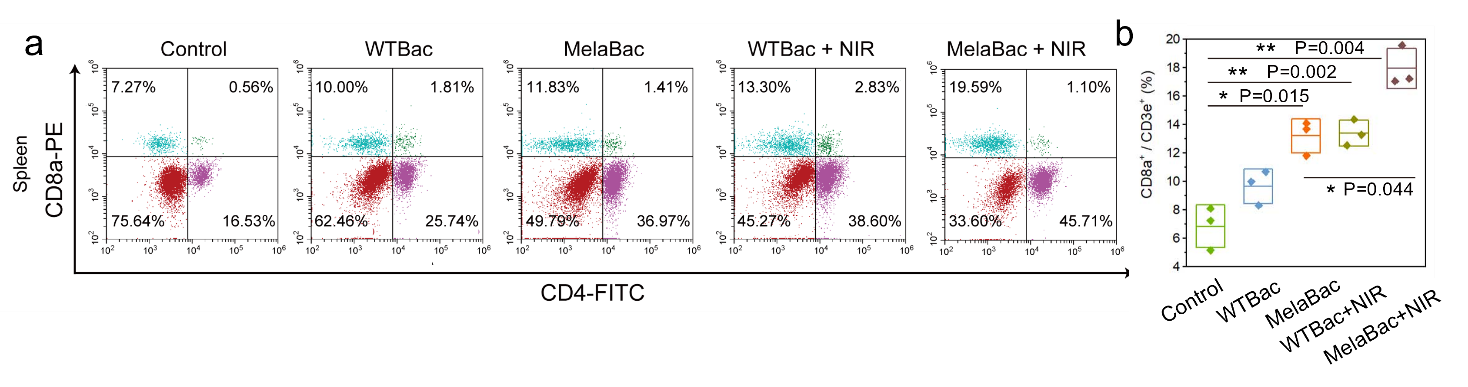


**Figure S21.** a-b, Flow cytometry analysis (a) and statistical analysis (b) of CD8+ T cells in CD3+ T cells isolated from spleen tissues of mice from different groups.


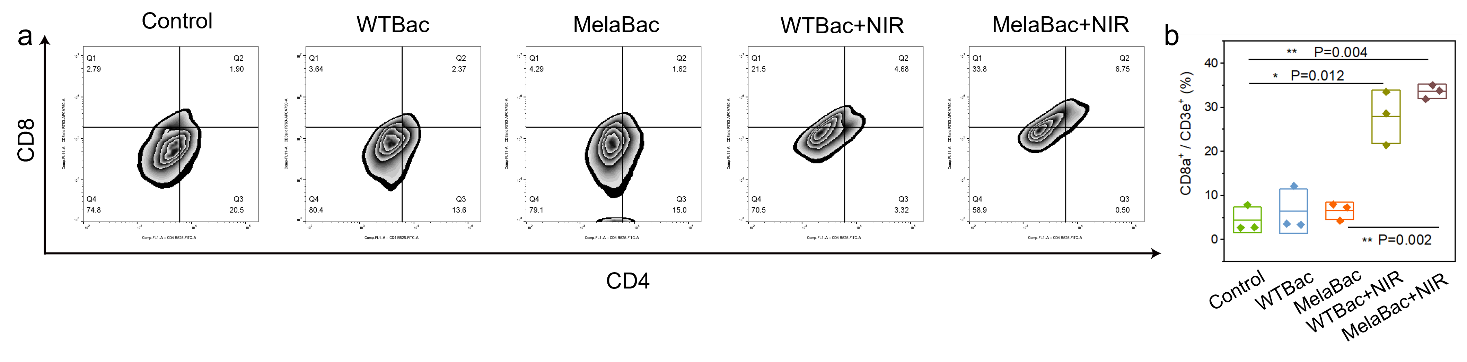


# Figure S22. a, Flow cytometry analysis of CD4/CD8 T cells isolated from tumor tissues in each group after varied treatments. b, Corresponding statistical analysis of CD8+ T cells in CD3+ T cells isolated from tumor tissues of mice from different groups.

#
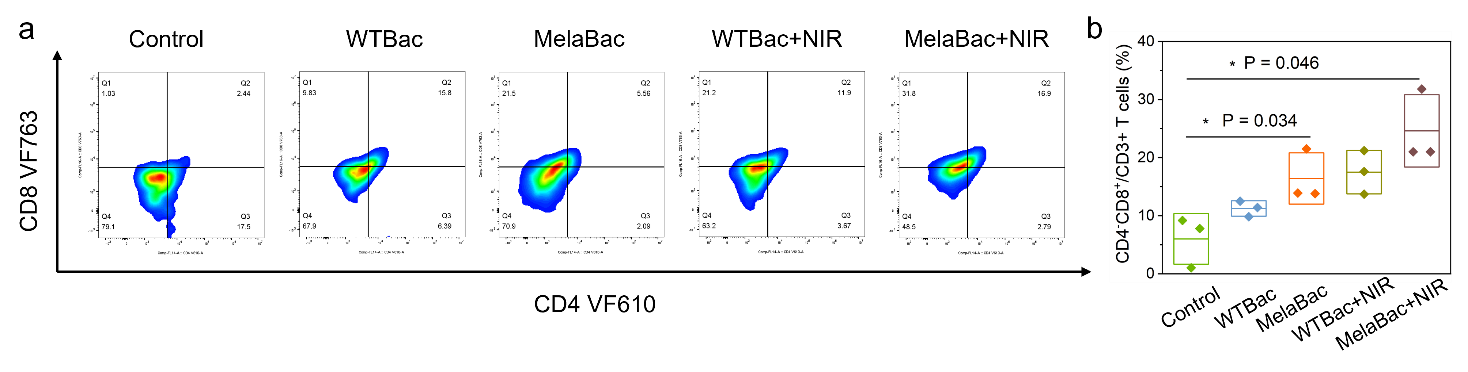


# Figure S23. a, Flow cytometry analysis of CD4/CD8 T cells isolated from lymph nodes of mice after varied treatments. b, Corresponding statistical analysis of CD8+ T cells in CD3+ T cells isolated from tumor tissues of mice from different groups.


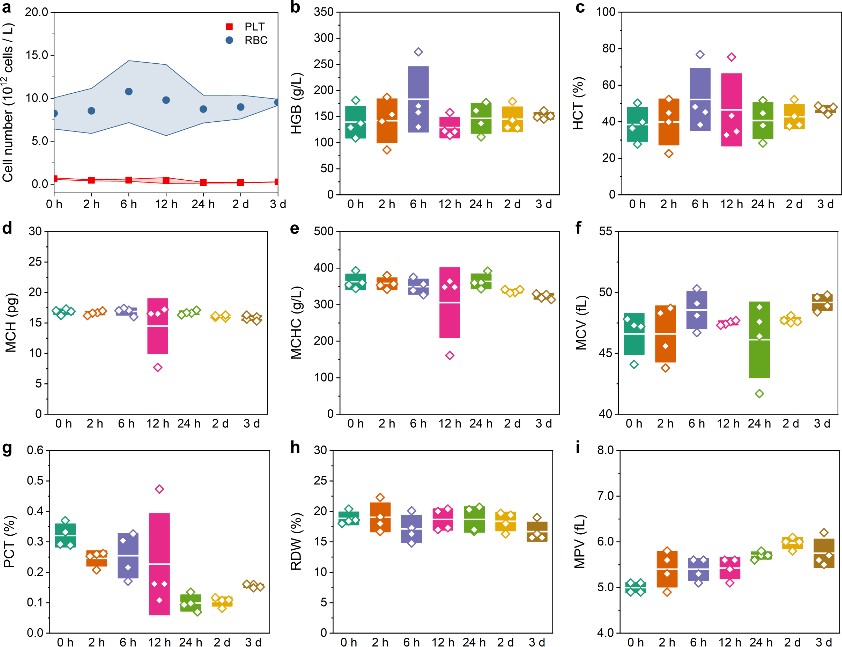


# Figure S24. Time-course blood routine analysis of mice administrated with MelaBac cells. Indices include (a) platelets (PLT) and red blood cells (RBC), (b) hemoglobin (HGB), (c) hematocrit (HCT), (d) mean corpuscular hemoglobin (MCH), (e) mean corpuscular hemoglobin concentration (MCHC), (f) mean corpuscular volume (MCV), (g) procalcitonin (PCT), (h) red cell distribution (RDW), and (i) mean platelet volume (MPV).


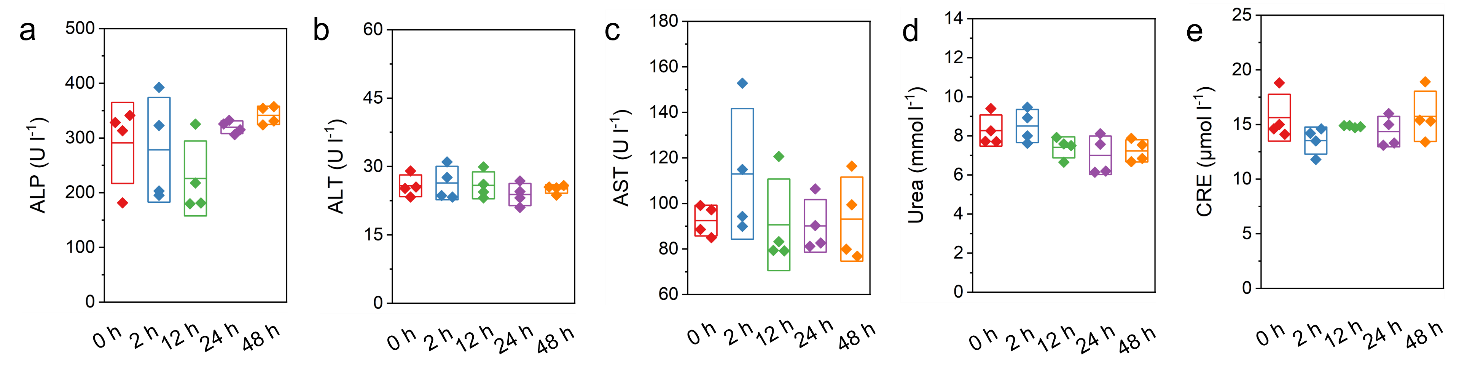


**Figure S25.** Time-course blood serum analysis ((a) alkaline phosphate (ALP), (b) alanine transaminase (ALT), (c) aspartate transaminase (AST), (d) urea and (e) creatine (CRE)) of mice administrated with MelaBac cells.


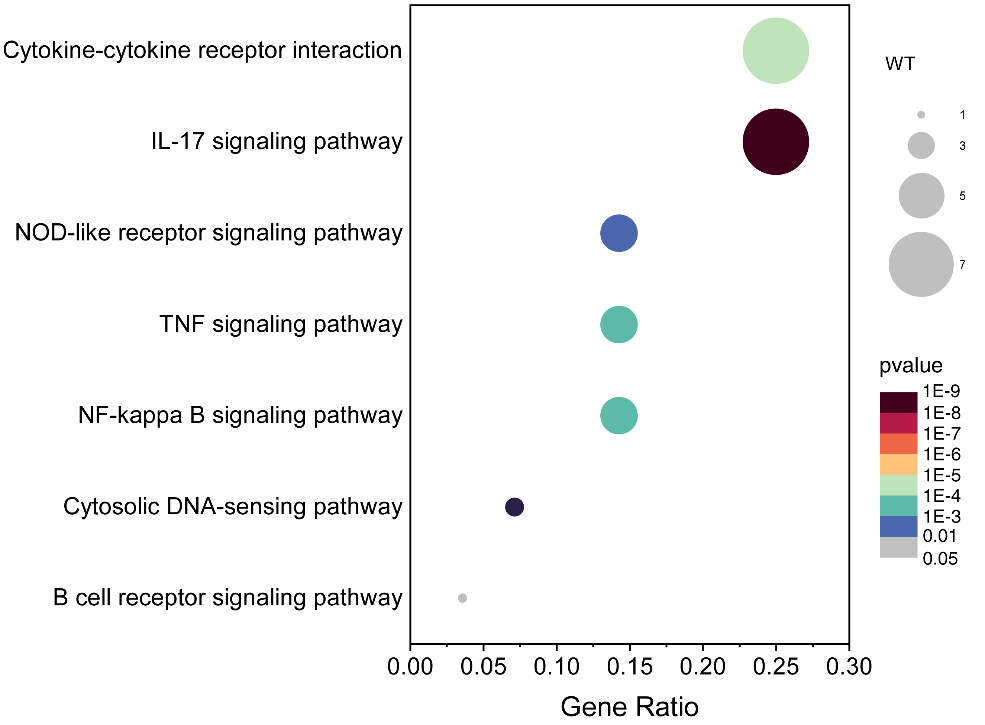


**Figure S26.** KEGG enrichment analysis of the differentially expressed genes between Control and WTBac group.


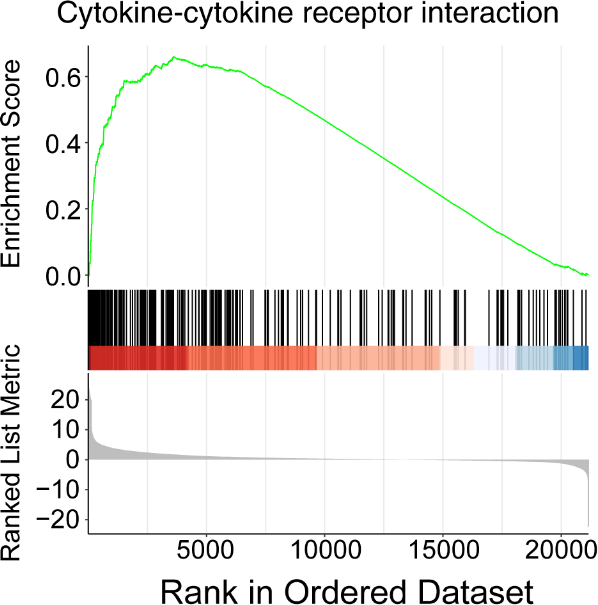


# Figure S27. GSEA image of cytokine-cytokine receptor interaction.


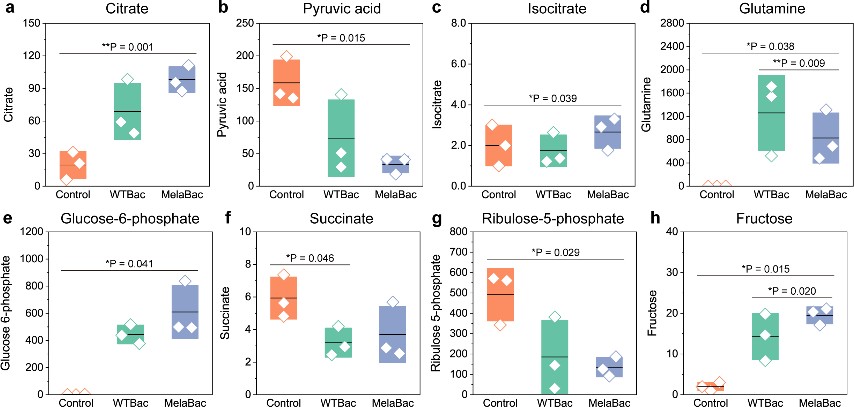


# Figure S28. Quantification and statistical analysis of metabolites: (a) citrate, (b) pyruvic acid, (c) isocitrate, (d) glutamine, (e) glucose 6-phosphate, (f) succinate, (g) ribulose 5-phosphate and (h) fructose of mice from different groups.


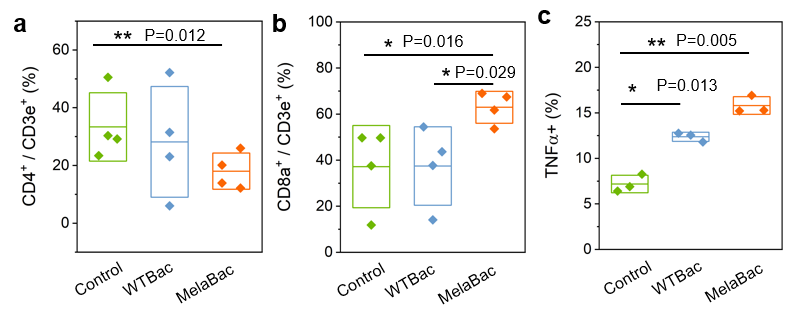


# Figure S29. a-c, Quantification and statistical analysis of the cell populations in the spleen of CD3+CD4+ (a), CD3+CD8+ (b) and TNFα+/CD4+ (c) percentage.


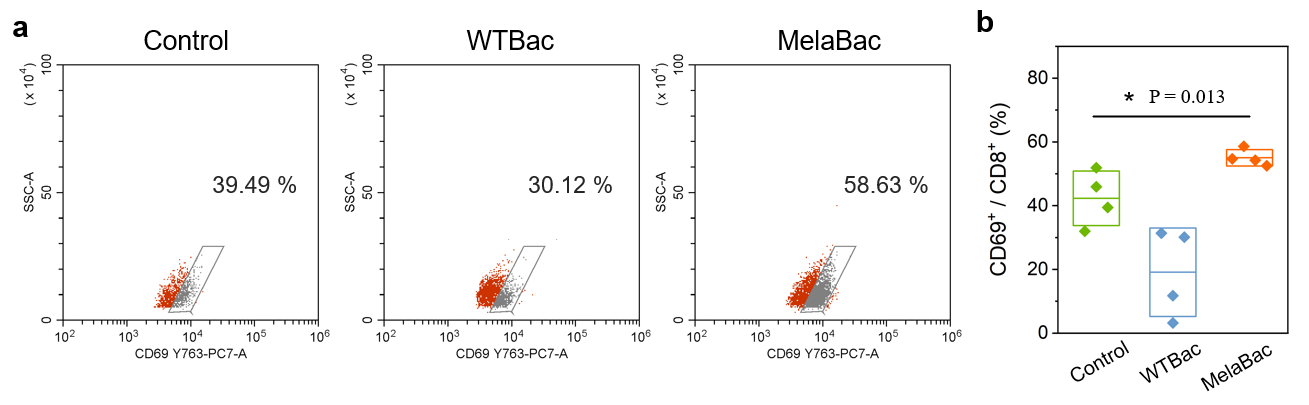


**Figure S30.** a-b, Flow cytometry analysis (a) and statistical analysis (b) of CD69+ T cells in CD8+ T cells isolated from spleen tissues of mice from different groups.

# Table S1.

Sequences of protein used in this study.

| Protein  Name | Protein Sequence | DNA Sequence |
| --- | --- | --- |
| TyrA | MTVRKNQASLTAEEKRRFVAALLELKRTGRYDAFVTTHNAFILGDTDNGERTGHRSPSFLPWHRRFLLEFERALQSVDASVALPYWDWSADRSTRSSLWAPDFLGGTGRSRDGQVMDGPFAASAGNWPINVRVDGRTFLRRALGAGVSELPTRAEVDSVLAMATYDMAPWNSGSDGFRNHLEGWRGVNLHNRVHVWVGGQMATGVSPNDPVFWLHHAYIDKLWAEWQRRHPSSPYLPGGGTPNVVDLNETMKPWNDTTPAALLDHTRHYTFDV | ATGACAGTCCGTAAAAATCAAGCATCCTTGACGGCGGAAGAGAAACGTCGTTTTGTAGCGGCATTACTTGAATTAAAACGTACCGGCCGTTATGACGCGTTCGTGACTACCCACAATGCATTCATTCTGGGGGATACCGATAACGGTGAACGCACAGGCCACCGCTCGCCCAGCTTTCTGCCCTGGCATCGCCGCTTCTTGTTGGAGTTTGAACGCGCACTGCAATCGGTCGATGCGAGCGTCGCCCTGCCGTATTGGGATTGGTCCGCGGATCGCTCAACCCGCTCTTCGCTGTGGGCGCCAGATTTTTTGGGCGGCACGGGCCGTAGCCGCGACGGCCAGGTTATGGATGGCCCTTTCGCAGCCTCGGCAGGTAATTGGCCGATTAATGTCCGTGTGGACGGCCGCACGTTCCTCCGCCGTGCGTTAGGCGCCGGCGTGAGCGAACTGCCAACCCGCGCTGAAGTGGACAGCGTCCTGGCCATGGCTACCTACGATATGGCGCCCTGGAACTCCGGCAGCGATGGCTTTCGCAATCATTTAGAGGGTTGGCGCGGTGTCAATCTGCATAACCGCGTGCACGTTTGGGTTGGTGGTCAAATGGCCACGGGCGTTAGCCCCAATGATCCGGTTTTCTGGTTACACCATGCCTATATTGATAAATTATGGGCGGAATGGCAGCGCCGTCATCCGAGCAGCCCGTATTTGCCAGGCGGAGGTACGCCCAACGTGGTGGACTTAAATGAAACTATGAAGCCGTGGAATGACACGACCCCCGCAGCCCTGTTAGATCATACCCGTCACTATACCTTTGATGTC |
| Cofactor | MPELTRRRALGAAAVVAAGVPLVALPAARADDRGHHTPEVPGNPAASGAPAAFDEIYKGRRIQGRTVTDGGGHHGGGHGGDGHGGGHHGGGYAVFVDGVELHVMRNADGSWISVVSHYEPVDTPRAAARAAVDELQGARLLPFPSN | ATGCCAGAACTCACACGCCGTCGCGCGCTGGGAGCTGCAGCGGTCGTGGCTGCGGGAGTCCCACTGGTGGCCCTCCCGGCCGCACGTGCAGACGATCGCGGGCACCATACGCCCGAAGTTCCGGGCAACCCAGCGGCATCGGGTGCCCCCGCCGCCTTTGATGAGATCTACAAGGGCCGCCGTATTCAGGGTCGTACTGTGACGGATGGCGGCGGACACCATGGAGGTGGTCATGGTGGTGATGGCCACGGCGGTGGTCACCACGGTGGCGGTTATGCGGTTTTTGTTGACGGGGTCGAGCTCCATGTAATGCGTAACGCGGATGGGAGCTGGATCTCGGTGGTGAGCCACTACGAACCGGTCGATACTCCTCGCGCGGCCGCGCGCGCAGCGGTAGATGAATTACAGGGAGCCCGCCTGCTCCCGTTCCCTAG  CAAT |

# Table S2.

Sequences of key blocks used in this study.

| Name | Type | DNA Sequence |
| --- | --- | --- |
| pJ23108 | Promoter | CTGACAGCTAGCTCAGTCCTAGGTATAATGCTAGC |
| SLP2018  -2-167 (p2800) | Promoter | GCGCCAGACAATTTCTGT ATTTGACAAATCTGATGC TACGAAGTATAATCTACT TCCGGCCCGGTTACGGTTAACGAA |
| SLP2018  -2-101 (p1400) | Promoter | GCTCTCGACAGAAATTCG TGTTGACACTCCAAGAAG GTCCTTTTATAATGATTAC CCCCATCGGGCATTTCCGGCGCT |
| rrnB T1 | Terminator | CAAATAAAACGAAAGGCT CAGTCGAAAGACTGGGCC TTTCGTTTTATCTGTTGTT TGTCGGTGAACGCTCTCCTGAGTAGGACAAAT |

**References**

1. Martin, M. (2011). Cutadapt removes adapter sequences from high-throughput sequencing reads. EMBnet. 17(1), 10-12.

2. Kim D, Langmead B, Salzberg SL. HISAT: a fast spliced aligner with low memory requirements. Nat Methods. 2015;12(4):357-360.

3. Pertea M, Pertea GM, Antonescu CM, Chang TC, Mendell JT, Salzberg SL. StringTie enables improved reconstruction of a transcriptome from RNA-seq reads. Nat Biotechnol. 2015;33(3):290-295.

4. Love MI, Huber W, Anders S. Moderated estimation of fold change and dispersion for RNA-seq data with DESeq2. Genome Biol. 2014;15(12):550.

5. Wu T, Hu E, Xu S, et al. clusterProfiler 4.0: A universal enrichment tool for interpreting omics data. Innovation (N Y). 2021;2(3):100141.
